# Organic geochemistry and mineralogy suggest anthropogenic impact in speleothem chemistry from volcanic show caves of the Galapagos

**DOI:** 10.1016/j.isci.2022.104556

**Published:** 2022-06-09

**Authors:** Ana Z. Miller, Nicasio T. Jiménez-Morillo, Mathilda L. Coutinho, Fernando Gazquez, Vera Palma, Francesco Sauro, Manuel F.C. Pereira, Fernando Rull, Theofilos Toulkeridis, Ana T. Caldeira, Paolo Forti, José M. Calaforra

**Affiliations:** 1 Instituto de Recursos Naturales y Agrobiologia de Sevilla (IRNAS-CSIC), Seville, Spain; 2 HERCULES Laboratory, University of Évora, Évora, Portugal; 3 MED—Mediterranean Institute for Agriculture, Environment and Development, University of Évora, Évora, Portugal; 4 Department of Biology and Geology, University of Almería, Almería, Spain; 5 Andalusian Centre for the Monitoring and Assessment of Global Change (CAESCG), University of Almería, Almería, Spain; 6 Department of Earth Sciences and Environmental Geology, University of Bologna, Bologna, Italy; 7 CERENA, Instituto Superior Técnico, University of Lisbon, Lisbon, Portugal; 8 CSIC-CAB Associated Unit ERICA, Department of Condensed Matter Physics, Mineralogy and Crystallography, University of Valladolid, Boecillo, Spain; 9 Universidad de las Fuerzas Armadas (ESPE), Campus Sangolquí, Sangolquí, Ecuador

**Keywords:** Earth sciences, Geochemistry, Geology, Volcano

## Abstract

The network of lava tubes is one of the most unexploited natural wonders of the Galapagos Islands. Here, we provide the first morphological, mineralogical, and biogeochemical assessment of speleothems from volcanic caves of the Galapagos to understand their structure, composition, and origin, as well as to identify organic molecules preserved in speleothems. Mineralogical analyses revealed that moonmilk and coralloid speleothems from Bellavista and Royal Palm Caves were composed of calcite, opal-A, and minor amounts of clay minerals. Extracellular polymeric substances, fossilized bacteria, silica microspheres, and cell imprints on siliceous minerals evidenced microbe-mineral interactions and biologically-mediated silica precipitation. Alternating depositional layers between siliceous and carbonate minerals and the detection of biomarkers of surface vegetation and anthropogenic stressors indicated environmental and anthropogenic changes (agriculture, human waste, and cave visits) on these unique underground resources. Stable isotope analysis and Py-GC/MS were key to robustly identify biomarkers, allowing for implementation of future protection policies.

## Highlights

• Speleothems from lava tubes of Galapagos are archives of anthropogenic stressors
• Moonmilk and coralloids are composed of calcite, opal-A, and clay minerals
• Microbe-mineral interactions promote mineral dissolution and precipitation
• Biomarkers of surface vegetation and anthropogenic impacts detected by Py-GC/MS

## Graphical abstract

**Figure:**
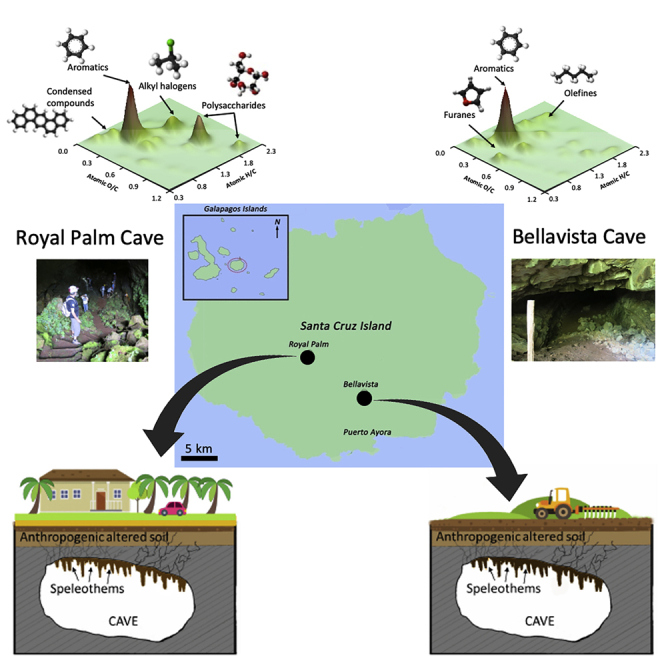

## Introduction

The Galapagos Islands (Ecuador), a volcanic archipelago in the Pacific Ocean and inextricably linked to Charles Darwin’s theory of evolution, epitomize unusual abiotic and biotic evolutionary processes because of their unique ecosystems and ongoing geological and geomorphological features. Regardless of the extraordinary biodiversity of the Galapagos, scientific interest in their geological features is tied to ongoing volcanic activity, including hot spot volcanism and plate tectonic interactions, as well as to the young geological age of the islands (between one million to five million years). These islands embrace lava structures of various ages resulting from repeated volcanic eruptions and depicting complete sequences of geological and geomorphological processes (Debut et al., 2021; Kempe et al., 2021).

Despite the increasing interest in speleology, the underground network of basaltic lava caves of pahoehoe lava flows is one of the most unexploited geological wonders of the Galapagos (Constantin et al., 2019; Gallardo and Toulkeridis, 2008; Taylor et al., 2011). The first speleological contributions date back to 1965, resulting from the “Mission Scientifique Belge aux Galapagos” expedition in 1962. In 1965, N. and J. Leleup conducted the first studies on cave biology, describing new species of terrestrial troglobite fauna (Hernández et al., 1992). However, research work on cave microbiology and geomicrobiological interactions is still neglected. To the best of our knowledge, the first microbiological study on Galapagos lava tubes was carried out by Miller et al. (2020b) who characterized the microbial diversity of moonmilk and coralloid speleothems from two lava tubes of Santa Cruz Island. During this speleological expedition, different types of hard and soft speleothems, such as moonmilk, crusts, micro-gours and botryoidal coralloids, were described (Daza et al., 2016; Miller et al., 2020b). These types of secondary mineral deposits, with specific morphological features, are frequently reported in lava tubes worldwide (Gonzalez-Pimentel et al., 2018; Maciejewska et al., 2018; Miller et al., 2014, 2015, 2018; Park et al., 2020)^.^

Moonmilk are whitish deposits with soft, cotton-like or spongy appearance, frequently found in karstic caves (Cañaveras et al., 2006; Hill and Forti, 1997; Maciejewska et al., 2015). Its mineralogy often comprises microcrystalline aggregates of carbonate minerals as major compounds (e.g., calcite, hydromagnesite, aragonite, vaterite, and huntite) (Cuezva et al., 2003; Hill and Forti, 1997), but minor amounts of nitrates and sulfates have also been identified (Martínez-Arkarazo et al., 2007). Calcite moonmilk is formed by aggregates of needle-shaped calcite crystals, also denominated needle-fiber calcite (NFC) (Stoops, 1976), with a wide range of morphologies (Miller et al., 2018). The origin of the NFC is still contradictory, with authors ascribing an abiotic (Borsato et al., 2000; Lacelle et al., 2004) or biotic origin (Cañaveras et al., 2006; Cuezva et al., 2003), or even a combination of both during different growth steps (Cailleau et al., 2009; Miller et al., 2018).

Coralloids are speleothems with botryoidal morphology, frequently recorded in lava tubes (Aubrecht et al., 2008; Urbani et al., 2005; Vidal-Romaní et al., 2010). The composition of these speleothems is mainly opal-A—a hydrated opaline amorphous silica. Other minerals, such as calcite and clay minerals (e.g., halloysite, sepiolite, etc.), have also been detected in coralloids (Miller et al., 2014; Webb and Finlayson, 1987; Wray, 1999). These speleothems rarely exceed a few centimeters because of the slow dissolution rate of silica under abiotic and temperature conditions (Gonzalez-Pimentel et al., 2018; Webb and Finlayson, 1987; White, 2010; Wray and Sauro, 2017). The processes driving their growth may be affected by surface conditions (e.g., precipitation, changes in temperature, airflows, vegetation, and soil cover), biological processes (e.g., subsurface bacteria) and influenced by anthropogenic activities (e.g., land use changes, cave visits, cave adaptation for tourists) (Baker and Genty, 1998). These environmental changes have a profound impact on cave systems, with significant alterations in speleothem growth, by modifying the amount and flow path of the water entering into the cave. Miller et al. (2016) showed for the first time that siliceous speleothems from Easter Island (Chile) recorded the well-known ecocide occurred on the island. The authors used Py-GC/MS and compound specific isotope analysis to infer on the origin of some organic components of the speleothems and to detect some indicators of surface environmental and land use changes. Recently, Miller et al. (2020a) reported that surface ecological alterations caused by wildfires changed speleothem formation and chemistry in a lava tube from La Palma Island (Spain). Siliceous speleothems from lava tubes are thus important archives of past environmental conditions and surface land use changes from volcanic regions (Miller et al., 2016, 2020a).

Here, we provide the first in-depth morphological, mineralogical, and organic geochemical characterization of moonmilk and coralloid-type speleothems from two lava tubes of Santa Cruz Island (Galapagos) to extrapolate on their biogenicity and identify biomarkers of environmental and anthropogenic changes recorded in these speleothems. Specifically, the structure and mineralogical composition of the speleothems were described using micro-computed tomography, scanning electron microscopy, infrared and Raman spectroscopy, and X-Ray diffraction analyses. Stable isotope analysis and analytical pyrolysis, in combination with graphical-statistical tools, were performed to assess the molecular composition of the organic fraction preserved in the speleothems.

## Results and discussion

### Morphological and mineralogical characterization of the speleothems

The speleothems analyzed in this study were collected in two lava tubes of Santa Cruz Island from the Galapagos (Ecuador) and classified as moonmilk and coralloid-type speleothems, based on their morphological characteristics.

### Moonmilk deposits from Bellavista Cave (Bella-M)

**Figure 1:**
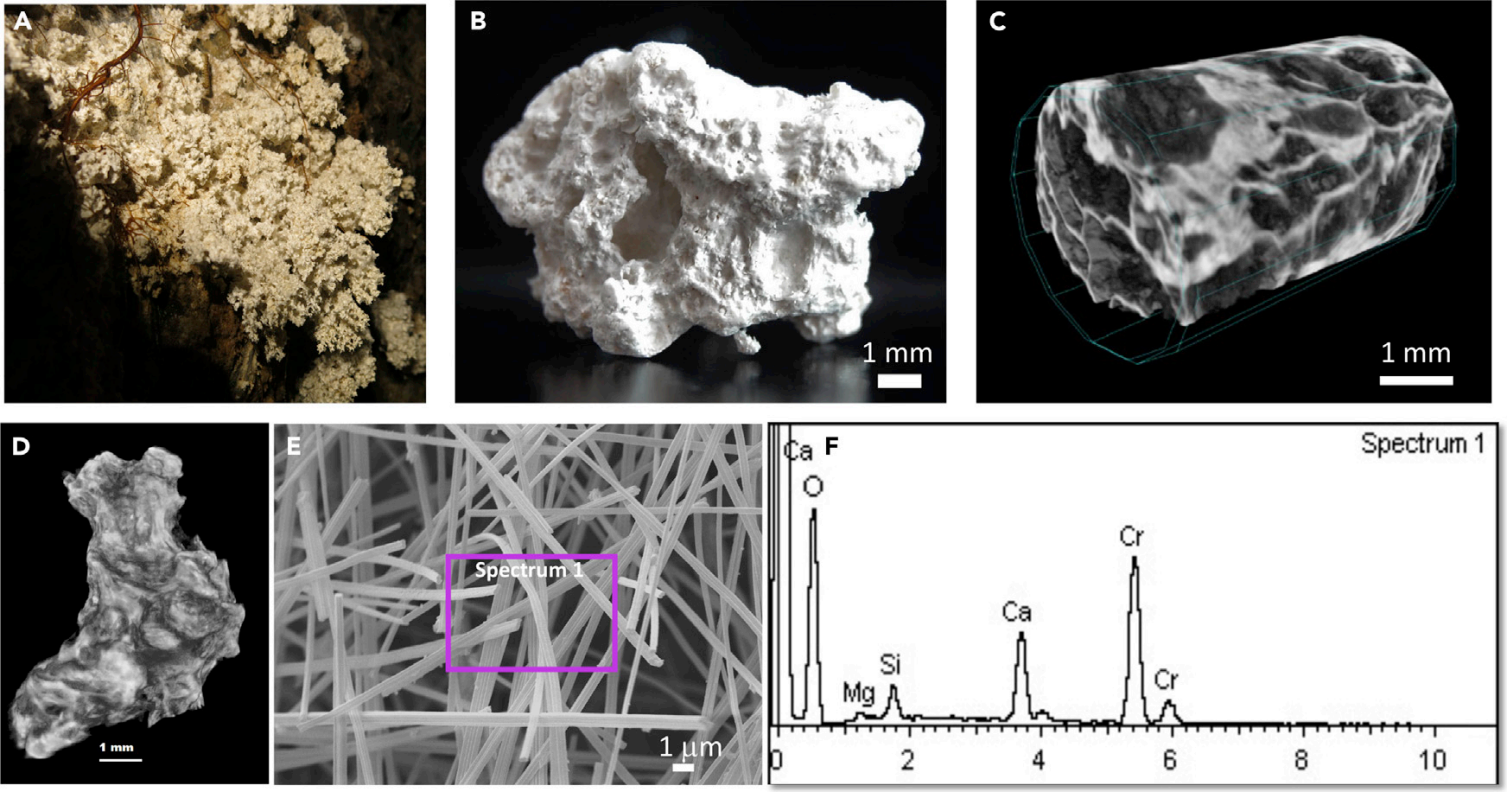
Moonmilk deposits (Bella-M) from Bellavista Cave (A) Field image from the moonmilk deposits with soft and arborescent texture; (B) Air-dried moonmilk showing white color and pasty texture; (C) micro-CT image of the internal structure of the moonmilk deposits; (D) X-ray micro-CT image of the external surface of the sample; (E) FESEM image showing the needle-shaped fibers, and (F) EDS spectrum obtained from the area marked on panel E (spectrum 1).

The whitish moonmilk deposits (designated Bella-M), with arborescent texture (Figure 1A), were collected from Bellavista Cave (Figure S1A), a 2316 m-long lava tube located in Bellavista (Kempe et al., 2021), an agricultural village 6 km away from the capital of Santa Cruz. When air-dried, the samples showed white color and a brittle, porous and pasty texture (Figure 1B), which is characteristic of moonmilk (Cañaveras et al., 2006; Miller et al., 2018). The micro-computed X-ray tomography (micro-CT) provided information on the shape and internal distribution of mineral phases according to their opacity to X-rays. The reconstructed radiographs of Bella-M showed a very porous and poorly consolidated structure, with dark areas corresponding to the presence of voids and light areas to denser materials, probably Ca-rich phases (Figures 1C and 1D).

Field Emission Scanning Electron Microscopy with Energy Dispersive X-ray Spectroscopy (FESEM-EDS) revealed intertwined clusters of needle-shaped fibers of different size (>20 μm long and <1 μm in diameter) and striated surfaces (Figure 1E). These fibers were composed of Ca with minor amounts of Mg and Si (Figure 1F). The crystal shape and elemental composition confirmed the presence of the needle-fiber calcite (NFC), characteristic of moonmilk deposits reported in limestone and granite subterranean environments (Cañaveras et al., 2006; Miller et al., 2018). Curiously, moonmilk from lava tubes has been scarcely reported and investigated (Gonzalez-Pimentel et al., 2021; Miller et al., 2020b; Polyak and Provencio, 2006).

Mineralogical analyses performed by X-ray diffraction (XRD) of Bella-M showed the characteristic patterns of calcium carbonate crystallized in the form of calcite (according to Powder Diffraction File number 00-005-0586) with sharp and well-defined peaks (Figure S2A). Vibrational spectroscopy studies using Fourier transform infrared (FTIR) and Raman techniques also evidenced the presence of calcite (Figures S3A and S4A and Table S1) (Parker et al., 2010). In addition, vibrational bands associated with silicates with tetrahedrally coordinated silicon were detected (Table S1). FTIR analysis also revealed a peak at about 669 cm^−1^ (Figure S3A), representing Mg–OH vibrations (Miller et al., 2014; Webb and Finlayson, 1987).

According to Raman analysis, traces of a silicate mineral probably of the smectite group, such as montmorillonite or saponite (Wang et al., 2015), were identified (Figure S4A and Table S1). A small broad band in the C-H stretching region (2800–3100 cm^−1^) was detected by Raman (Figure S4A), which could be related to the presence of organic compounds (Figure S4A and Table S1) (Howell et al., 1999).

### Coralloid-type speleothems from Bellavista Cave (Bella-C)

**Figure 2:**
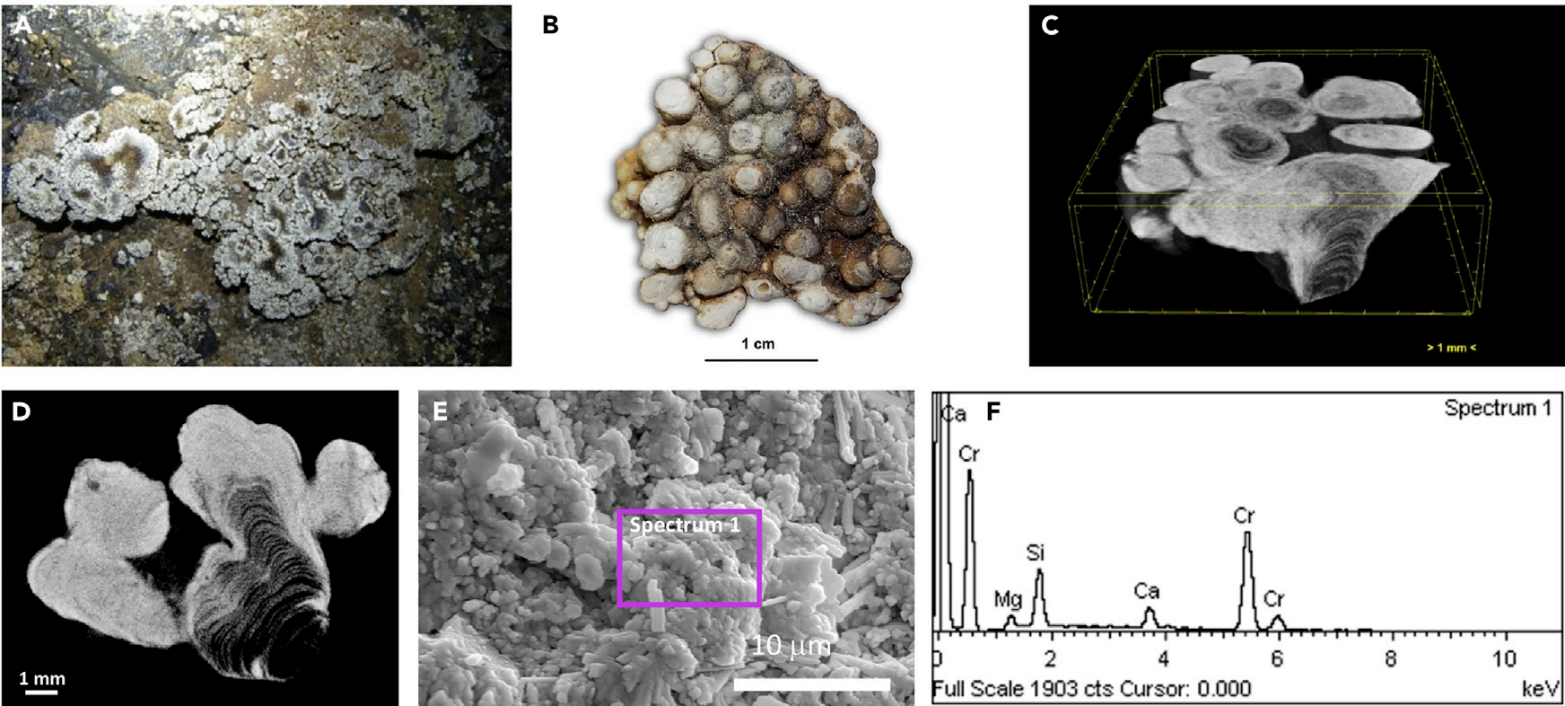
Coralloid speleothems (Bella-C) from Bellavista Cave (A) Field image from the white coralloids on the cave floor; (B) Air-dried coralloid sample showing globular texture; (C and D) micro-CT images of the internal structure of the coralloids; (E) FESEM image showing heterogeneous texture, and (F) EDS spectrum obtained from the area marked on panel E (spectrum 1).

Whitish to light gray speleothems (designated Bella-C) were also found and collected in the touristic trail of Bellavista Cave (Figure S1A), the oldest show cave from Santa Cruz Island. They consist of globular-shaped coralloids developed on the floor of the cave, covering approximately 50 m of the cave passage, at the end of the lava tube (Figure 2A). Under the stereomicroscope, Bella-C samples were cauliflower-like coralloids, growing upwards from the substrate, composed of globular units with ∼0.5 cm in diameter, and gray to brownish in color (Figure 2B). The micro-CT radiographs and respective reconstructed slices showed an internal structure composed of a succession of very thin layers in the innermost part of the globular forms, with abundant semitransparent products to X-ray (darker areas, Figures 2C and 2D) separated by submillimeter opaquer layers (light gray areas, Figure 2D). The FESEM-EDS showed a heterogeneous surface (Figure 2E), with variable proportions of Si, Mg and Ca (Figure 2F), reinforcing the micro-CT data. The Si and Mg enriched domain was more transparent to X-ray radiation (dark areas), suggesting the presence of an amorphous siliceous and magnesium silicate mineral phase, whereas the Ca-rich phase (bright regions in micro-CT images) probably corresponds to crystalline calcite.

The diffractogram of Bella-C coralloids pointed to magnesian calcite (Figure S2B; PDF card number 00-043-0697) explaining the presence of Mg in the EDS microanalysis. In comparison with Bella-M, the diffractogram of Bella-C showed broader peaks because of the lower crystallinity of the carbonates.

The FTIR analysis of Bella-C showed strong bands in the Si-O stretching region, typical of framework silicates (Sodo et al., 2016), and a band at 794 cm^−1^, which has been considered a distinguishing feature of opal-A (Sodo et al., 2016). Some vibrational bands linked to calcite were also reported by FTIR and Raman (Figures S3B and S4B and Table S1). Carbonate-siliceous coralloid speleothems, with alternating layers showing different mineralogical composition, were also reported in lava tubes (López-Martínez et al., 2016; Miller et al., 2014), limestone caves (Suchý et al., 2021) and granite cavities (Vidal-Romaní et al., 2010). This mineralogical intercalation during speleothem growth may occur because of changes on surface environmental conditions or land use, or to internal factors within the cave, such as changes in pH, in the chemical composition of the dripping water or adaption works of the cave to tourism accessibility (Miller et al., 2016, 2018; 2020a).

### Coralloid speleothems from Amor Tunnel (Amor-C)

**Figure 3:**
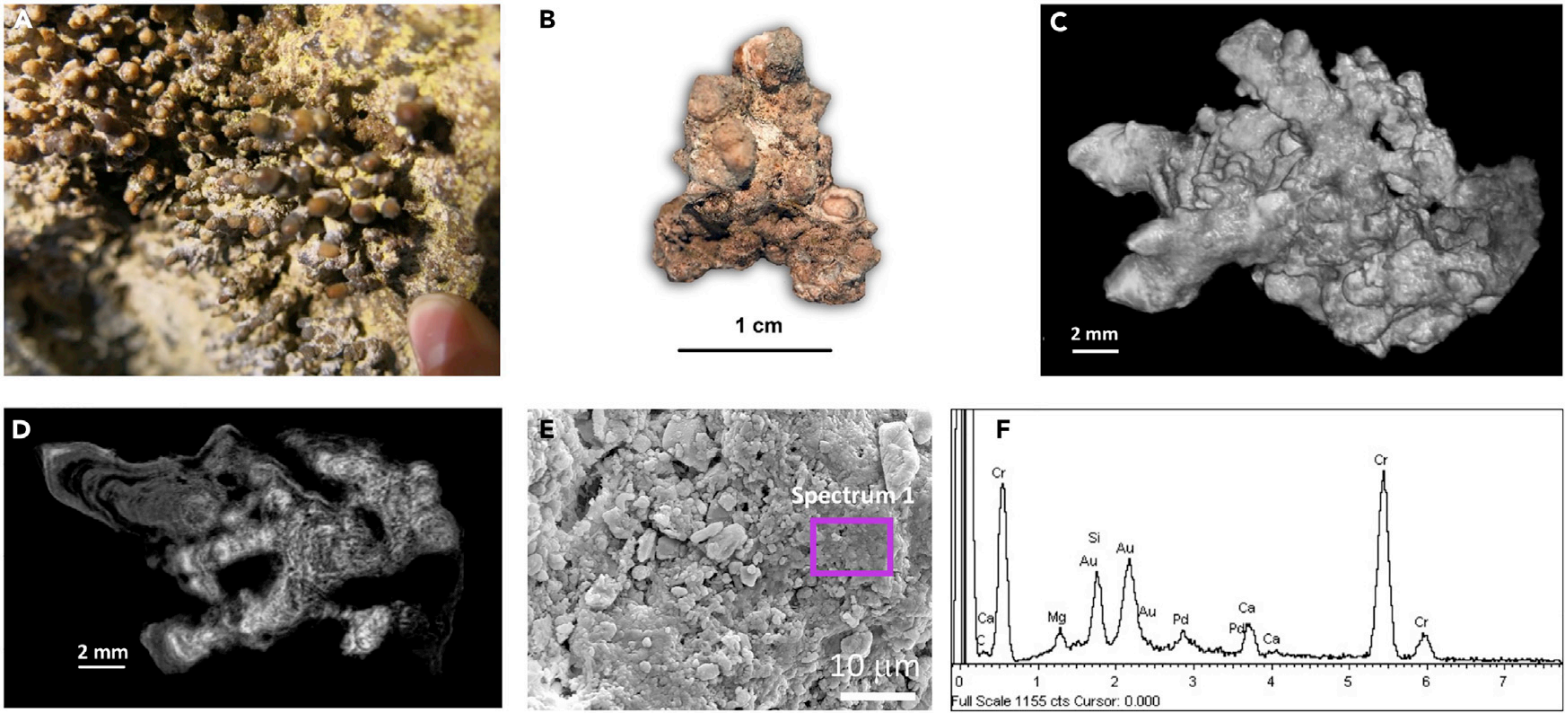
Brownish coralloid speleothems (Amor-C) from Amor Tunnel (A) Field image from the coralloids on the cave wall; (B) Air-dried sample showing branched coralloid; (C and D) micro-CT images of the internal structure of the coralloid sample; (E) FESEM image of the surface of the coralloids showing heterogeneous texture, and (F) EDS spectrum obtained from the area marked on panel E (spectrum 1).

Brownish-colored coralloid stalactites (designated Amor-C), coated with yellow microbial mats (Figure 3A), were collected in Amor Tunnel, another touristic section of Bellavista Cave (Figure S1A). Under high magnification, Amor coralloids were branched and irregular in shape, with rough external surfaces (Figure 3B). Each branch ranged from less than 2 mm to about 5 mm in diameter, and from 0.5 cm to approximately 2 cm in length (Figures 3B and 3C). In addition, thin white crusts were often visible on the surface of the coralloids (Figure 3B).

The internal structure was concentric banded, composed of white opaque domains, with alternating semiopaque gray and dark transparent phases, as revealed by micro-CT (Figure 3D). FESEM-EDS observations of Amor-C corroborated micro-CT results, showing a heterogeneous surface topography rich in Si with a minor amount of Mg (Figures 3E and 3F).

The XRD pattern of Amor-C samples (Figure S2C), showed a broad diffuse band centered at about 21.7° 2θ (4.1 A), characteristic of opal-A (Aubrecht et al., 2008; Miller et al., 2014; Sodo et al., 2016; Webb and Finlayson, 1987). Peaks assigned to magnesian calcite were also found (Figure S2C). Vanghi et al. (2017) reported low magnesium calcite in coralloids from Lamalunga Cave (Italy), probably present in solution at the time of coralloids formation. The Mg incorporation in speleothem calcite from karstic caves is expected when Mg/Ca molar ratio is >0.3 and <1.5 in the parent water (Davis et al., 2004; Richter et al., 2011). In this case, water samples were not collected, precluding inferences on the source of Mg found in the speleothems from the Galapagos lava tubes.

FTIR spectra of Amor-C coralloids showed the silicate framework vibrations and low intensity bands of calcite (Figure S3C and Table S1) (Dovbeshko et al., 2004). As reported in Bella-M, a strong band in the C-H stretching region was also observed, which could be related to the presence of organic compounds (Table S1).

### Coralloid speleothems from Royal Palm Cave (Royal-C)

**Figure 4:**
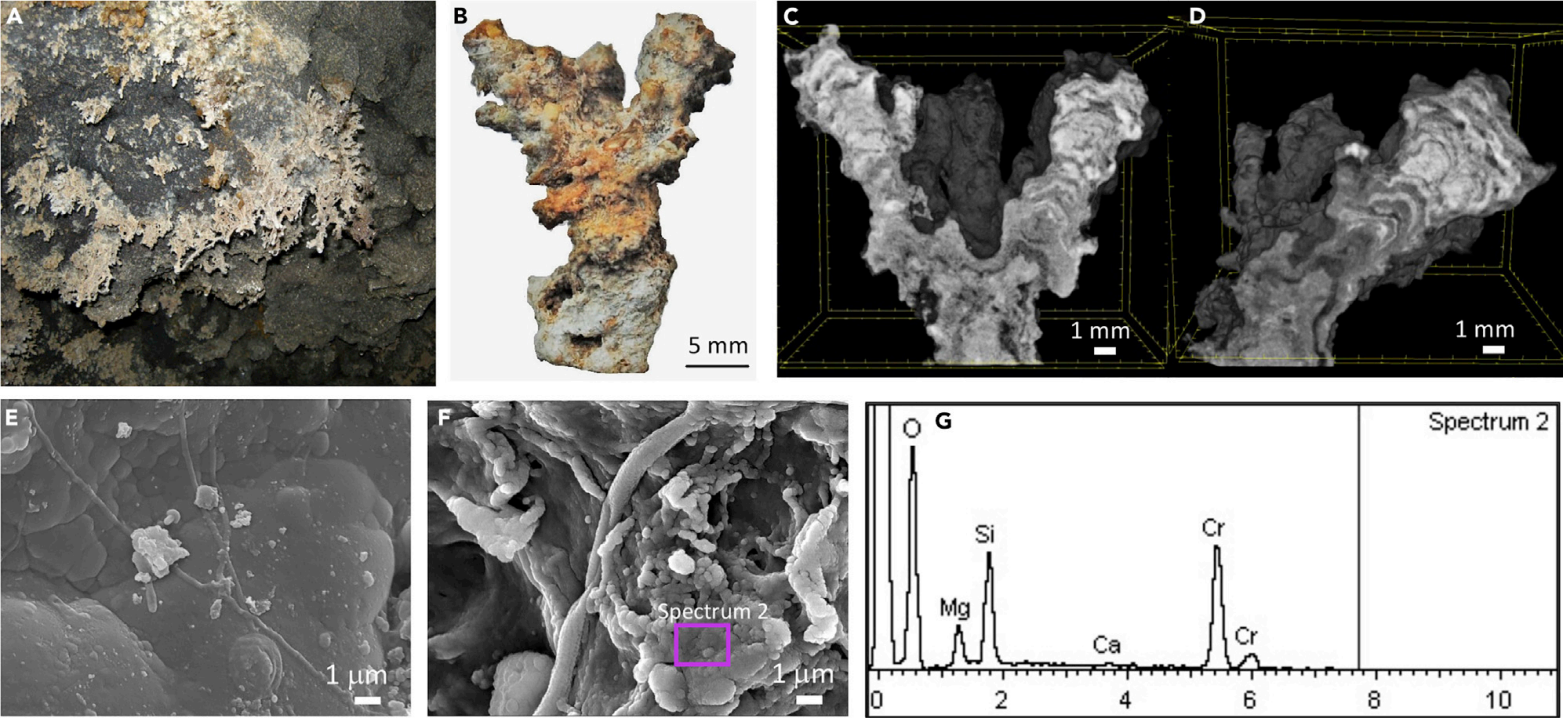
Coralloid speleothems (Royal-C) from Royal Palm Cave (A) Field image from the coralloids; (B) Air-dried coralloid sample depicting gray to orange color and white coatings; (C and D) micro-CT images of the internal structure of the coralloids showing internal layering with different composition, mainly Ca-rich minerals; (E,F) FESEM images showing botryoidal structure, and (G) EDS spectrum obtained from the area marked on panel F (spectrum 2).

Grayish coralloids (designated Royal-C) were collected from the ceiling of Royal Palm Cave (Figure S1B). It is a 1040 m lava tube managed by the Royal Palm Hotel (Kempe et al., 2021), located in the western part of Santa Cruz Island, near Santa Rosa Village, adjacent to the Galapagos National Park. The Royal coralloid speleothems were irregular-shaped branched stalactites of small size, with only a few centimeters in length and less than 0.5 cm in diameter (Figures 4A and 4B). These speleothems displayed light gray to orange color, and a rough external surface with whitish coatings (Figure 4B). The micro-CT study revealed a complex internal structure mainly characterized by variations in material X-ray absorption. The reconstructed radiographs showed internal compositional zonation along the growth direction of the coralloids, evidencing mineralogical changes during speleothem growth (Figures 4C and 4D). The first stage of mineral deposition was dominated by alteration products, possibly of siliceous composition, more transparent to radiation (gray areas, Figure 4D), whereas the opaquer products, usually Ca-rich minerals, were dominant in the final stage of speleothem growth (bright areas, Figure 4D). Miller et al. (2016) showed that different colored layers from coralloid speleothems of a lava tube in Easter Island (Chile) were related to different stages of speleothem formation caused by different water regimes and an increase in the average temperature during speleothem growth. The micro-CT models of Royal-C showed a general increase of opaque products (Ca-rich phases) in the outermost part of the speleothems, suggesting changes in the environmental conditions during the final stage of speleothem formation.

FESEM-EDS of the bulk coralloid samples revealed a botryoidal structure with smooth rounded surfaces, characteristic of opal-A (Miller et al., 2014) (Figure 4E). EDS microanalysis conducted on a cluster of botryoidal particles showed they were composed of O, Si, and Mg (Figures 4F and 4G). The Si/O ratio (in weight %) was close to 0.50, which is typical of pure silica and opal. However, the enrichment in Mg suggests the presence of sepiolite, a hydrous magnesium silicate clay mineral, which was recorded in opal coralloids from Mt. Hamilton Cave (Australia) (Webb and Finlayson, 1987) and Ana Heva lava tube (Chile) (Miller et al., 2014). In some areas, small amounts of Ca were also detected (Figure 4G).

For Royal-C samples, vibrational spectroscopy analysis by FTIR and Raman showed spectral features that can be related to clay minerals, opal-A and calcite (Figures S3D and S4D and Table S1). In addition, C-H vibrations of organic compounds, previously reported in Bella-M and Amor-C, were also detected for Royal-C samples (Table S1).

### Microbe-mineral interactions

**Figure 5:**
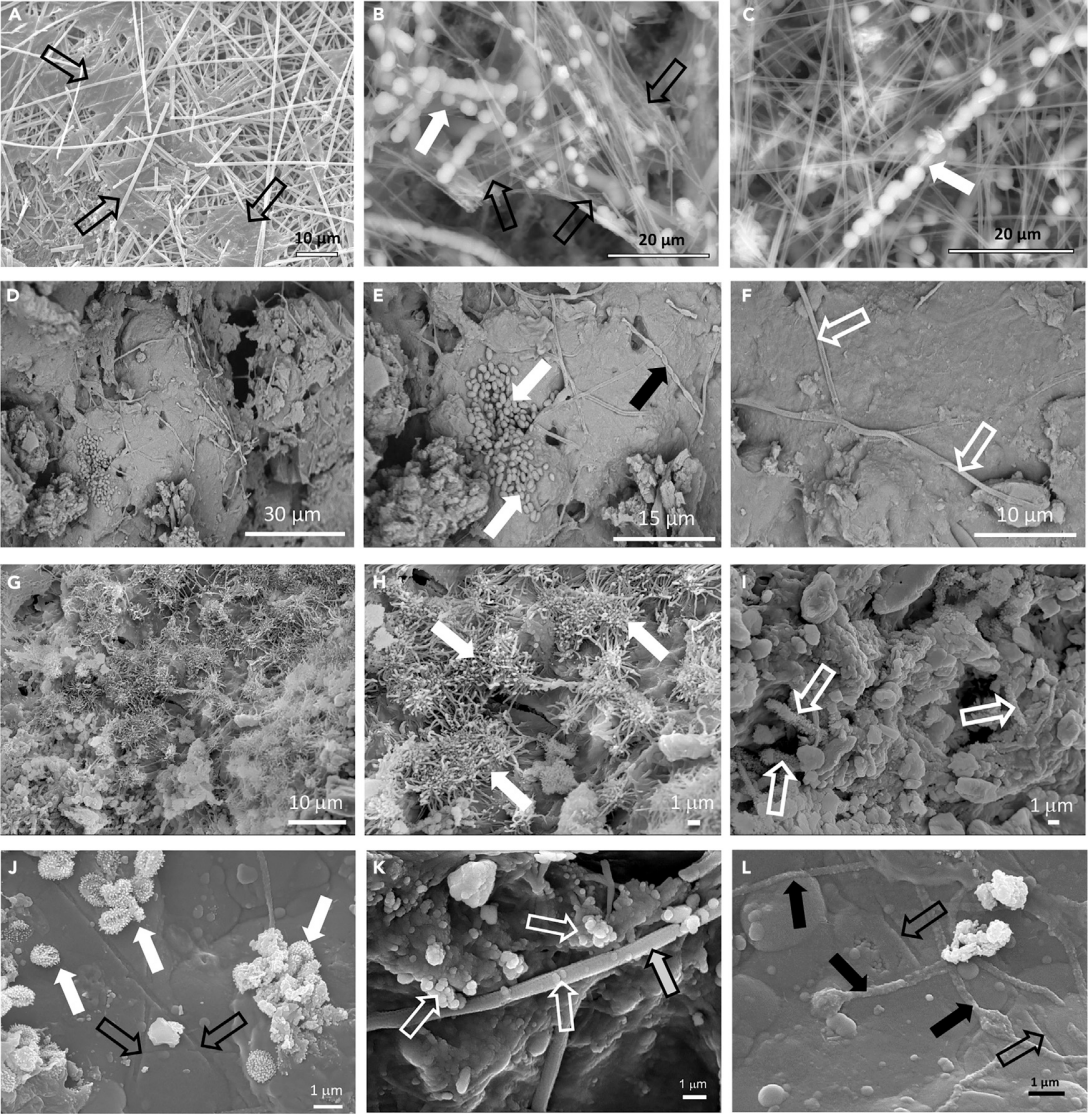
FESEM images of the speleothems collected from lava tubes of Santa Cruz Island (Galapagos, Ecuador) showing microbe-mineral interactions (A–C) Bella-M moonmilk samples depicting intertwined needle-fiber calcite (NFC) embedded in extracellular polymeric substances (open black arrows) and wrapped in coccoid-shaped cells (white arrows); (D–F) Bella-C coralloid speleothems showing a complex microbial consortium composed of bacillary forms (white arrows), chains of rod-shaped cells (black arrow) and reticulated filaments (open white arrows), embedded in EPS (open black arrows); (G–I) Amor-C speleothems displaying tangled mass of hyphae, EPS and spores of Actinobacteria-like structures (white arrows), and bacillary-shaped cells with spiny ornamentation (open white arrows); (J–L) Royal-C coralloids showing Actinobacteria-like coccoid cells with spiny ornamentation (white arrows), reticulated filaments (gray arrow) and other silicified filamentous structures (Figure 5L). In addition, imprints of microbial filaments are observed on the siliceous substrate (open black arrows).

FESEM examinations were conducted to assess microbe-mineral interactions, to recognize biosignatures and to infer on the biogenicity of the studied speleothems. The moonmilk deposits Bella-M showed the characteristic NFC (Figures 5A–5C). These fibers were found in association with a matrix of extracellular polymeric substances (EPS) and microbial cells (Figures 5A–5C), as previously reported by Miller et al. (2020b) These fibers were wrapped in coccoid-like microbial cells with smooth surfaces, resembling a string of pearls (Figures 5B and 5C). Each spherical bioform measured 3–4 μm in diameter and linked together to form large linear aggregates, some of them resembling binary fission (Figure 5B). Similar features were reported by Suchý et al. (2021) in carbonated-siliceous coralloids from Koněprusy caves system (Czech Republic). These bioforms were found embedded in a slimy matrix of EPS, which can provide nucleation sites for the precipitation of minerals, such as NFC (Cañaveras et al., 2006; Cuezva et al., 2012; Miller et al., 2012; Riquelme et al., 2015). Miller et al. (2018) suggested a biogenic origin for the initial stage of NFC, as Sanchez-Moral et al. (2012) and Cacchio et al. (2014) did, followed by an abiotic process. Our FESEM observations suggest an association between microbial activities and the formation of the NFC, evidenced by the presence of a slime matrix of EPS and coccoidal microbial cells intimately associated with NFC (Figures 5A–5C). The carbon isotope composition of the carbonate fraction of Bella-M (−10.3‰) also indicated a biotic origin of the NFC.

The FESEM images of the coralloid speleothems from Bellavista Cave (Bella-C) revealed the presence of microbial structures of different shapes and sizes, mainly filamentous and rod-shaped forms, embedded in a matrix of EPS forming biofilms (Figures 5D–5F). Most of the microbial features observed on Bella-C samples comprised: (i) clusters of bacillary forms with >1 μm in length (Figure 5E), (ii) chains of rod-shaped cells (Figure 5E, white arrows), resembling *Streptomyces*-like arthrospores (Riquelme et al., 2015), and (iii) long filamentous forms with 0.5 μm in diameter and >50 μm in length, similar to the reticulated filaments reported by Melim et al. (2008) and Miller et al. (2012) These filaments were widely observed on the surface of the coralloid speleothems in association with EPS (Figure 5F, arrows). Reticulated filaments have been frequently reported in caves worldwide and have been associated with biomineralization of opal-A in lava tubes (Miller et al., 2014), calcium in limestone caves (Jones, 2009; Melim et al., 2008), and manganese oxides in a granite spring water tunnel (Miller et al., 2012). Yet, they could not be affiliated to any known microorganism, as most of these filaments are found as hollow mineralized sheaths (Miller et al., 2012). Rapid fossilization of microorganisms and microbial mats by mineral replacement preserving their morphology is a common process, indicative of biogenicity (Westall et al., 2006). Recently, Melim et al. (2015) reported living reticulated filaments in a limestone cave in Germany, showing the presence of carbon on the filaments without replacement by minerals.

The coralloid speleothems from Amor Tunnel (Amor-C) showed several organic structures (Figures 5G–5I). Microbial features were mainly found as a biofilm consortium formed by a tangled mass of hyphae and spores of Actinobacteria-like structures (Figures 5G and 5H), with a variety of surface spore ornamentation, including hairy (Figure 5G) and spiny (Figure 5I) ornamentation. Actinobacteria are ubiquitous in subsurface environments, including lava tubes (Riquelme et al., 2015), and have been previously identified by DNA-based analysis in speleothems from Bellavista Cave (Miller et al., 2020b). They grow forming colored microbial mats coating siliceous speleothems, as observed in Galapagos lava tubes, and promoting biomineralization processes (Gonzalez-Pimentel et al., 2018; Miller et al., 2020b; Riquelme et al., 2015).

The FESEM-EDS of Royal-C also showed abundant microbial structures and EPS associated with the coralloids from Royal Palm Cave (Figures 5J–5L). Microbial features consisted of Actinobacteria-like coccoids with spiny ornamentation (Figure 5J), reticulated filaments (Figure 5K) and other silicified filamentous structures (Figure 5L). The surface of the reticulated filaments was covered with opal spheres with <0.5 μm in diameter, and clusters of spheroidal opal bodies were observed on the EPS matrix. The close relationship between Si-rich botryoidal clusters and reticulated filaments, including their EPS, as well as their similar morphology to the biogenic coralloid speleothems found in a lava tube from Easter Island (Miller et al., 2014), suggest that biogenic opal-A is present in Royal Palm Cave. Other filamentous structures with mineralized sheaths were also observed in Royal-C (Figure 5L).

Definitive morphological evidence of microbe-mineral interactions comprise: i) mineralized structural microbial components with their characteristic sizes and shapes; ii) amorphous phases in close association with microbial cells and biofilms regulating mineral grain size and habit; iii) etching patterns or imprints produced by actively metabolizing cells on mineral substrate because of the excretion of organic acids (Miller et al., 2014; Riquelme et al., 2015; Westall et al., 2006, 2015). All of these morphological features support the biologically-mediated precipitation of silica forming the coralloid speleothems from the Galapagos lava tubes. The botryoidal texture of silica microspheres embedded in EPS indicates that biofilms acted as nucleation sites for opal precipitation. Nucleation of amorphous silica onto the surfaces of microorganisms have been documented in laboratory (Amores and Warren, 2007), lava tubes (Miller et al., 2014), quartzite caves (Sauro et al., 2018), and hot spring environments (Handley et al., 2008; Jones and Renaut, 1996).

Other lines of evidence of microbe-mineral interactions in the speleothems from the studied Galapagos lava tubes were cell-sized filamentous imprints on the silicified substrate of Royal-C samples (Figures 5J and 5L). The presence of etched minerals associated with microorganisms evidences mineral dissolution by organic acids. Microbial dissolution of siliceous substrates is a common process, especially in oligotrophic environments such as lava tubes because of the survival strategy of extremophiles to acquire essential elements (Westall et al., 2015). This microbial dissolution process contributes to weathering of Earth’s continental crust and pedogenesis (Napieralski and Roden, 2020). Evidence of mineral dissolution induced by microorganisms comprise cell-sized etch pits, micro-boring, and tunnel-like structures, as well as imprints of microbial filaments on mineral grains (Riquelme et al., 2015). Many of these microbe-mineral interactions, largely comprising mineral dissolution and precipitation, result in the preservation of traces of microbial features or metabolic activity in the rock record. These traces of microbial life have recently received much attention because they have been recognized as biosignatures valuable for astrobiology (Northup et al., 2011; Westall et al., 2015).

### Isotope and molecular composition of the speleothems

The carbon and nitrogen isotope composition of the organic fraction of the moonmilk and coralloid speleothems are shown in Table 1. The δ^13^C and δ^15^N values showed significant statistical differences among the caves. However, within the same cave, no significant differences (p > 0.05) in terms of ^13^C and ^15^N composition were observed (Bella-M and Bella-C). The δ^13^C value of these samples ranged between the mean value of C_3_ and C_4_ plants (Fry, 2006). Bellavista Cave is located within an agricultural region, where the main plantation is the banana-tree (*Musa paradisiaca*). This is a C_3_ plant, but maize (*Zea mays*) and cassava (*Manihot esculenta*) also grow in this region (Barrera et al., 2021), which are typical C_4_ plants. This mixture of crops might explain the isotope values found in the organic fraction of Bellavista speleothems. Previous studies on siliceous speleothems from lava tubes showed a direct correlation between the aboveground vegetation, and the carbon isotope composition of organic matter in speleothems (Miller et al., 2016; 2020a). On the other hand, the molecular alteration of the organic matter in speleothems by microorganisms (microbial reworking) may generate ^13^C-enrichment. Jiménez-Morillo et al. (2020a; 2020b) observed a significant enrichment of the heavy carbon isotope in soil fine fractions, which evidenced microbial activity.

**Table 1:** Mean (±standard deviation) carbon and nitrogen stable isotope values of the organic fraction from the speleothem samples of Galapagos lava tubes, with one-way ANOVA

Sample ID	δ^13^C (‰, VPDB)	δ^15^N (‰, V-Air)
Bella-M	−15.7 ± 0.8 c	2.9 ± 0.5 b
Bella-C	−12.7 ± 0.9 c	3.5 ± 1.5 b
Amor-C	−26.5 ± 0.0 a	−5.4 ± 0.6 a
Royal-C	−17.3 ± 0.3 b	11.1 ± 1.5 c

The different letters indicate significant (*p* < 0.05) differences between samples (Tukey's test).

The agricultural contribution in Bella samples can be also supported by the low δ^15^N values, which were close to 0‰, generally corresponding to the signature of inorganic fertilizers (Szpak, 2014). This indicates an input of anthropized agricultural material from the overlying layers.

In the case of Amor-C, collected in the Amor Tunnel from Bellavista Cave, the δ^13^C value was characteristic of C_3_ plants (−26.5‰). This demonstrates the direct influence of surface vegetation overlying caves in speleothem chemistry (Miller et al., 2016; 2020a). The lowest δ^15^N concentration (−5.4 ± 0.6‰) was obtained for Amor-C, which also supports the contribution of aboveground vegetation runoff. Gerschlauer et al. (2019) found negative isotope values of nitrogen in natural rainforest biomass from Mount Kilimanjaro. On the other hand, the presence of denitrifying microbial communities, previously identified by Miller et al. (2020b) in Bellavista Cave (e.g., *Vicinimibacteraceae*, *Iamiaceae*), could generate ^15^N-depletion (Granger and Wankel, 2016). These bacterial families are capable of transforming the nitrogen cycle in the cave environment (Miller et al., 2020b; Zhang et al., 2019). Hathaway et al. (2014) reported that ammonia-oxidizing bacteria and nitrogen-fixing bacterial populations from lava caves of the Terceira Island (Azores, Portugal) are affected by surface land use.

The intermediate carbon isotope composition of the organic fraction of Royal-C (−17.3 ± 0.3‰) may be explained by either the contribution of a mixture of C_3_ and C_4_ plants or the isotope fractionation by microbial activity (Miller et al., 2020b). The highest δ^15^N value observed in Royal-C suggests significant anthropogenic alteration of speleothems, as this nitrogen isotope composition is characteristic of human residues (fecal waters) (Kuhnle et al., 2013). This hypothesis is also supported by the presence of the pathogenic bacteria *Ralstonia pickettii* and *Shigella sonnei*, previously identified in Royal-C samples (Miller et al., 2020b), and found in fecal samples from Galapagos finches (Michel et al., 2018). Royal Palm Cave is a show cave managed by the Royal Palm Hotel for touristic use. The visits and works conducted for adapting the cave for tourists (wooden walkway and light system), as well as the potential leaking of fecal waters could explain these data.

**Figure 6:**
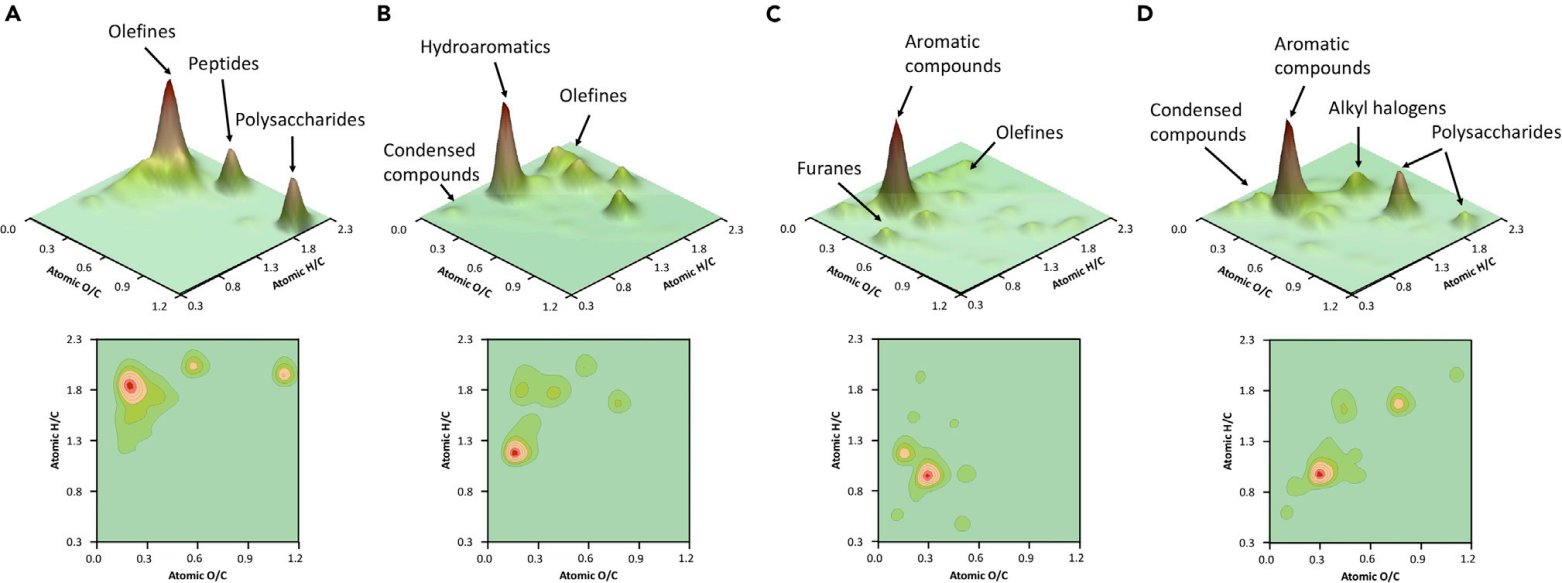
3D and contour van Krevelen diagrams of the moonmilk and coralloid-type speleothems of Galapagos lava tubes The figure shows the H/C and O/C ratios obtained by Py-GC/MS from the speleothem samples of the Galapagos lava tubes: (A) Bella-M; (B) Bella-C; (C) Amor-C, and (D) Royal-C.

The carbon and nitrogen stable isotope analysis of the speleothems from the Galapagos lava tubes revealed the existence of a significant organic fraction preserved in the rock samples. The molecular characterization of the organic compounds recorded in Bella-M by Py-GC/MS (Figure 6A) showed a highly diverse composition, mainly comprising compounds derived from lipids and proteins. These compounds are biomarkers of microorganisms (Jiménez-Morillo et al., 2016, 2018; 2020a; 2020b), which reinforces the existence of microbial life associated with the moonmilk deposits and their biogenic origin. Recently, Miller et al. (2020b) reported the presence of different microbial communities dominated by *Acidobacteria* and *Gemmatimonadetes*, in Bella-M. An exhaustive analysis of the organic molecules released by analytical pyrolysis from Bella-M revealed the presence of a relatively high proportion of linear, branched, and unsaturated alkyl-like compounds (Table S2). In addition, a noticeable influence of polysaccharide-derived compounds (H/C > 1.5 and O/C > 0.7) was observed (Figure 6A), probably derived from the copious amount of EPS excreted by microorganisms to form biofilms, and clearly observed by FESEM (Figures 5A–5C). The *Vicinimibacteraceae* family, previously reported by Miller et al. (2020b) in the Bella-M sampling site, are chemoorganotrophic bacteria, which produce a broad range of organic compounds, such as polysaccharides and organic acids, to grow in volcanic caves (Huber et al., 2016; Vieira et al., 2017). The existence of these compounds may indicate the presence of two well-differentiated organic carbon pools, one from subsurface microbial activity and another from lixiviation of surface plant biomass (Miller et al., 2016; 2020a).

The 3D van Krevelen plot of Bella-C (Figure 6B) revealed a relative high contribution of hydroaromatic (branched benzene) and low condensation (branched naphthalenes) organic compounds (H/C < 0.5, O/C < 0.1). These molecules may derive from: i) the microbial alteration of fresh plant tissues (Baldock et al., 1990; Hatcher et al., 1983) that may generate residual compounds with significant chemical and biological stability (Rabbi et al., 2014), or ii) the presence of pyrogenic organic compounds (combustion of plant biomass or fossil fuels) (Miller et al., 2020a), which may be altered by specific microbial communities. Recently, Miller et al. (2020a) have found condensed organic compounds in jelly-like speleothems from a lava tube in La Palma Island (Spain), derived from the fire-affected overlying soil. Liu et al. (2011) observed that brown fungi were able to degrade plant tissues producing highly stable and condensed compounds. In fact, some condensed organic compounds (naphthalene, naphthalene, 1,2-dihydro-6-methyl-, naphthalene, 1-methyl-, naphthalene, 2,3-dimethyl-, naphthalene, 2,3,6-trimethyl, and Chamazulene, Table S2) were detected, which are highly resistant to microbial degradation (Jiménez-Morillo et al., 2020a, 2020b; Kramer et al., 2004). Diketopiperazine compounds (i.e. cycled amino acids) were also detected in Bella-C coralloids, which may be used as markers (biosignatures) of microbial communities (Borgman et al., 2019; Perzborn et al., 2013).

The Amor-C coralloid speleothems displayed a complex organic matter fraction, mainly composed of a mixture of labile (furanes) and recalcitrant (aromatic and condensed) compounds (Figure 6C). FESEM observations of Amor-C showed abundant EPS associated with the speleothems (Figures 5G–5I), which may be the source of furane compounds. A noticeable influence of aromatic compounds (0.9 > H/C < 1.5; 0.2 > O/C > 1.0) was also observed, which can derive from defunctionalization of methoxyphenols (Jiménez-Morillo et al., 2018). These compounds are considered biomarkers of higher plants (Jiménez-Morillo et al., 2018, 2020a, 2020b; Sutton and Sposito, 2005). This result is supported by the δ^13^C and δ^15^N values obtained for Amor-C. In addition, the presence of lipid and protein-like compounds was observed to a lesser extent in Amor-C, indicating that the influence of microorganisms was lower than in Bellavista samples (Bella-M and Bella-C). The detailed analysis of the organic compounds released by analytical pyrolysis (Table S2) showed the existence of molecules derived from the use of fertilizers (*N*-(Dimethylthiophosphinyl)-3-aminopyridine) (Zulkarami et al., 2011), as well as from the degradation of polycondensed materials (e.g., branched naphthalenes). Previous molecular biology analysis has showed the presence of microorganisms in Bellavista Cave (e.g., *Gemmatimonadetes*, Proteobacteria, Firmicutes) (Miller et al., 2020b) capable of degrading polycyclic aromatic compounds (PAHs) (Mohapatra and Phale, 2021). The phylum *Gemmatimonadetes* was well-represented in Bella-M, with members assigned to the genera *Gemmatimonas* and *Roseisolibacter*, found in hydrocarbon-contaminated soils and subsurface environments (Miller et al., 2020b).

The molecular composition of the organic fraction of Royal-C speleothems, collected from Royal Palm show cave, was clearly marked by aromatic compounds, as well as polysaccharides (Figure 6D). In addition, there was a clear contribution of alkyl halogen and condensed-like compounds. Aromatic/condensed compounds were also detected by Raman (Figure S7 and Table S1). These recalcitrant molecules may be originated by the chemical and biological alteration of organic matter (Jiménez-Morillo et al., 2018), by ancient wildfire events (Miller et al., 2020a) or by the deposition of char residues probably generated by biomass combustion. Interestingly, DNA-based analysis of moonmilk and collaroid deposits in both caves showed the presence of *Advenella kashmirensis* and *Phenylobacterium haematophilum* bacteria (Miller et al., 2020b), which may obtain energy from the degradation of recalcitrant organic compounds. On the other hand, cleaning products, paintings and dissolvents may be the original source of alkyl halogen compounds, which are ubiquitous industrial chemicals widely recognized as persistent organic pollutants (Li et al., 2021). These findings suggest a significant anthropogenic impact in the subterranean environment, inducing changes on speleothem chemistry. The abundant presence of a bacterium capable of performing photosynthesis, *Gemmatimonas phototrophica*, in Royal Palm Cave (Miller et al., 2020b) also supports this hypothesis and evidences the impact of cave adaptation works for tourism purposes, such as the electric lighting system installed inside the cave (Figure S5). Tourist fruition of caves requires artificial lighting for both visitor safety and exhibition purposes. However, it is well-known that the light system promotes ecological alterations in the subterranean environment, particularly through the development of lampenflora (Burgoyne et al., 2021), as observed in Royal Palm Cave. These environmental and anthropogenic changes promote alterations on the growth and composition of speleothems. Thus, identifying most of the likely anthropogenic stressors (e.g., tourism, agriculture, and human habitation) threatening these subterranean environments represents a crucial step toward robustly examining key impacts and implement future geoheritage protection policies, as proposed by the cave conservation roadmap (Wynne et al., 2021).

### Limitations of the study

In this study, we identified biomarkers of anthropogenic stressors recorded in speleothems from two lava tubes of Santa Cruz Island (Galapagos, Ecuador). Comparisons with above lying soils, infiltration waters, and bedrock samples would be valuable to confirm the source of these organic compounds.

## STAR★Methods

### Key resources table

**Table:** 

REAGENT or RESOURCE	SOURCE	IDENTIFIER
**Software and algorithms**		

X’PERT High Score Plus	Malvern Panalytical	https://www.malvernpanalytical.com/en/products/category/software/x-ray-diffraction-software/highscore-with-plus-option
Powder Diffraction File 2	International Center for Diffraction Data	https://www.icdd.com/pdf-2/
RRUFF project database	RRUFF Project	https://rruff.info/
Qtegra ISDS	Thermo Fisher Scientific	https://www.thermofisher.com/order/catalog/product/IQLAAEGABSFAOVMBCZ
ChemStation	Agilent	https://www.agilent.com/en/product/software-informatics/analytical-software-suite/chromatography-data-systems/openlab-chemstation
Statgraphics Centurion XVI	Statgraphics	statgraphics.com/centurion-xvi

**Other**		

Shimadzu GC2010	Shimadzu	https://www.shimadzu.com/an/products/gas-chromatography/gas-chromatograph/gc-2010-pro/index.html
Shimadzu GCMS-QP2010 Plus	Shimadzu	https://www.shimadzu.com/an/products/gas-chromatograph-mass-spectrometry/single-quadrupole-gc-ms/gcms-qp2010-se/index.html
EGA/Py 3030D	Frontier Lab	https://www.frontier-lab.com/products/multi-functional-pyrolysis-system/17811/
Flash 2000 HT	Thermo Fisher Scientific	https://www.thermofisher.com/pt/en/home/industrial/mass-spectrometry/isotope-ratio-mass-spectrometry-irms.html
ConFlo IV	Thermo Fisher Scientific	https://www.thermofisher.com/pt/en/home/industrial/mass-spectrometry/isotope-ratio-mass-spectrometry-irms.html
Delta V Advantage IRMS	Thermo Fisher Scientific	https://www.thermofisher.com/pt/en/home/industrial/mass-spectrometry/isotope-ratio-mass-spectrometry-irms.html
micro-CT SkyScan 1172	Bruker	https://www.odont.uio.no/iko/english/about/organization/units/biomaterials/Capacities/%C2%B5ct-SkyScan-1172%20/
Jeol JSM-7001F FESEM	Jeol	https://www.jeol.co.jp/en/products/detail/JSM-7001F.html
X’PERT-PRO	PANalytical	https://www.malvernpanalytical.com/es/products/category/x-ray-diffractometers
Perkin Elmer Spectrum 65	Perkin Elmer	https://resources.perkinelmer.com/corporate/content/relatedmaterials/productnotes/prd_spectrum65.pdf
BWTEK Prime T BTC661E-785CUST	BWTEK	https://bwtek.com/
Finnigan MAT253	Thermo Fisher Scientific	http://www.thermo.com.cn/resources/200802/file_28763.pdf
GasBench II	Thermo Fisher Scientific	https://www.thermofisher.com/order/catalog/product/IQLAAMGAATFAETMBMZ
Philips XL40 SEM	Philips	https://www.mems-exchange.org/equipment/E1142/
EDS-EDAX 9900	EDAX	https://www.edax.com/contact-us

### Resource availability

#### Lead contact

Further information and requests should be directed to and will be fulfilled by the lead contact, A. Z. Miller (anamiller@irnas.csic.es).

#### Materials availability

This study did not generate new materials.

### Experimental model and subject details

Our study does not use experimental models.

### Method details

#### Studied site and sampling

The Galapagos Islands are an archipelago of 15 major volcanic islands, located in the eastern Pacific Ocean, about 1000 km west of the Ecuadorian coast. The first islands of the archipelago formed four to five million years ago due to sea bottom volcanic activity (Simkin, 1984). This region is considered one of the most active volcanic areas of the world with several active volcanos (Gallardo and Toulkeridis, 2008).

The climate of this region can be described as subtropical (Köppen climate classification: BSh), with average temperature of 27 °C during warm/wet (January-May) season and average temperature of 21° (June-December) in the cool season. The average annual rainfall is 575 mm, which is concentrated in the warm season (Climate data of Galápagos, 2021).

Santa Cruz Island, previously known as the Indefatigable Island, is in the east-central part of the archipelago and has an area of 986 km^2^, of which 88% of its territory belongs to the Galapagos National Park (Violette et al., 2014). This island is among the eldest Galapagos volcanoes Harpp et al. (2014), with lava ages ranging between 1.6 Ma to 30 ka (Schwartz, 2014; White et al., 1993).

A comprehensive speleological campaign, involving microbiology, mineralogy and geochemistry sampling approaches, was conducted in 2014 in lava tubes from Santa Cruz and Isabela Islands (Daza et al., 2016). A detailed description on the sampled lava tubes is provided in Daza et al. (2016) and Miller et al.(2020b). In the present study, moonmilk and coralloid-type speleothems collected in Bellavista Cave (Bella-M, Bella-C), Amor Tunnel (Amor-C) and Royal Palm Cave (Royal-C) from Santa Cruz Island were studied and analyzed.

Bellavista Cave, also known as “Gallardo Cave”, was discovered in 1948 by the schoolteacher Antonio Gallardo. The tourist sector of the cave is ∼1000 m (Figure S1A), and regular visits started in the 1960s, fitted with electric light since the 1980s. On average, 38 tourists enter the cave per day, i.e. 13,877 visitors per year, between 2000 and 2019 (T. Toulkeridis, personal communication).

The Royal Palm Cave is a show cave managed by the Royal Palm Hotel, with visits restricted to hotel guests (∼500 visitors/year). It has a wooden walkway and electric lighting comprising incandescent bulbs (Figure S5) along the entire length of the cave (T. Toulkeridis, personal communication). The chemicals employed in the upkeep of the wooden pathway are unknown.

Small fragments, but representative speleothem samples were taken in four different sampling points inside of Bellavista and Royal Palm Caves (Bella-M, Bella-C, Amor-C and Royal-C; Figure S1). Six composite replicates (*N* = 6) from each sampling site were collected within approx. 0.5 m radius using sterile tools (scalpels or chisels and hammer) and inserted directly into sterile 50 mL tubes.

#### Morphological, structural and mineralogical characterization

Morphological and structural characterization of the speleothems was carried by stereomicroscopy, field emission scanning electron microscopy (FESEM) and X-ray micro-computed tomography (micro-CT). Fragments of the speleothems were examined under a stereomicroscope Olympus SZ51 for macroscopic characterization. Subsequently, small fragments of the four speleothem samples (moonmilk and coralloids) were air-dried, directly mounted on SEM sample stubs and sputter coated with a thin conductive chromium (Cr) and/or gold (Au) film. A Jeol JSM-7001F FESEM, equipped with an Oxford X-ray energy dispersive spectroscopy (EDS) were used with secondary electrons mode and an acceleration voltage of 15 kV. The morphological characteristics of the speleothems were also examined using a Philips XL40 SEM coupled to an EDS-EDAX 9900 at 15 kV.

For the microstructural characterization of the speleothems, digital radiographs were acquired with a micro-CT SkyScan 1172 (Brucker) instrument using an X-ray cone incident on a rotating specimen. The instrument has a 1.3 Megapixel camera and can reach spatial resolutions of 5 μm with a detectability of 2 μm. Experimental details are described in Gonzalez-Pimentel et al. (2018).

The mineralogical composition of the speleothems was determined by X-ray diffraction (XRD) of random powder mounts, using a XPERT-PRO (PANalytical) diffractometer with CuKα radiation at 40 kV, 35 mA, 0.002° 2*θ* step size and 20 s of counting time. For phase analysis and determination, the analytical software X’PERT High Score Plus and the Powder Diffraction File 2 (PDF2) database were used.

Samples were also analyzed by Fourier Transform Infrared (FTIR) spectroscopy using a Perkin Elmer Spectrum 65 spectrometer for samples dried at 50°C to eliminate the effect of water molecules. Powdered samples were dispersed in KBr pellets and IR spectra were recorded for the 4000 cm^−1^ to 400 cm^−1^ regions.

Raman analyses of powdered samples were performed using a BWTEK BRM-OEM-785 diode laser (785 nm) and a BWTEK BAC100-785E Raman head. A BWTEK Prime T BTC661E-785CUST spectrometer with a Hamamatsu CCD (S10141-1107S, 2048 pixels) was used. The equipment covers a spectral range in Raman displacement of 0–3000 cm^−1^, with a spectral resolution of 5 cm^−1^. The Raman probe used a 20× lens to produce an 85 μm diameter laser spot on the sample. For each analysis, a maximum laser power of 140 mW was used, with a mean integration time of 2 s and 30 accumulations. The spectra of the RRUFF project database (https://rruff.info/) were used for mineralogical identification.

#### Biogeochemical characterization

The light carbon and nitrogen isotope composition (δ^13^C and δ^15^N, respectively) of the organic fraction of the speleothems were analyzed by elemental analysis/isotope ratio mass spectrometry (EA/IRMS) following the protocols described in Miller et al. (2016, 2020a). The δ^13^C and δ^15^N signatures were analyzed using a Flash 2000 HT with two reactors (combustion and pyrolysis). The EA peripherical is coupled by a ConFlo IV interface unit to a continuous flow Delta V Advantage IRMS (Thermo Scientific, Bremen, Germany). Isotope ratios are denoted by the delta (δ) notation in variations relative to the international standards, Vienna Pee Dee Belemnite (VPDB) for carbon and Vienna-Air (V-Air) for nitrogen, recognized by the International Atomic Energy Agency (IAEA). The carbon and nitrogen isotope composition is reported in the standard notation (‰). The standard deviation of bulk δ^13^C, and δ^15^N were ±0.1 and 0.3‰, respectively. Each replicate sample was measured in duplicate (*n* = 2). The removal of the inorganic fraction was carried out by sample digestion with a strong acid (HCl 1*M*) before EA analysis.

To determine the isotopic composition of inorganic carbon from the moonmilk calcite (Bella M), the moonmilk deposits were crushed to a fine powder and flushed with CP grade helium in Exetainer tubes (Labco®), then acidified with 104% H_3_PO_4_ and left to react for 1h at 70°C. Carbon isotopes of CO_2_ were measured using a Thermo Scientific GasBench II, equipped with a CTC autosampler coupled to a MAT253 mass spectrometer in the Godwin Laboratory at the University of Cambridge, UK. Analytical precision was estimated at ±0.03‰ for δ^13^C by repeated analysis of an inhouse Carrara Marble standard.

The molecular characterization of the organic fraction preserved in the speleothems was conducted by analytical pyrolysis coupled to gas chromatography/mass spectrometry (Py-GC/MS), as described elsewhere (Jiménez-Morillo et al., 2020a). Briefly, approximately 20 mg of each speleothem sample (*n* = 6) was introduced into crucible capsules and pre-heated for 1 min in a micro-furnace at 500°C for pyrolysis. The compounds evolved were then directly injected into a Shimadzu GC2010 gas chromatographer, which was equipped with a Phenomenex Zebron-ZB-5HT capillary column, and Shimadzu GCMS-QP2010 Plus mass selective detector. The mass spectra were acquired at 70 eV ionizing energy in negative mode. Organic compounds identification was attained via single-ion monitoring (SIM) and by comparison with mass spectra libraries (NIST14 and Wiley7).

The isotope and organic molecular characterization were discussed here based on the microbial diversity previously studied by Miller et al. (2020b) in Bellavista and Royal Caves.

### Quantification and statistical analysis

One-way analysis of variance (ANOVA) with Tukey’s post hoc testing was performed using the Statgraphics Centurion XVI statistical package to evaluate statistical differences in the C and N stable isotope values. From the structural information provided by the pyrolysis analysis, it was possible to construct 3D surface and contour van Krevelen diagrams using a home-made graphic-statistical software as described by Jiménez-Morillo et al. (2018).

## Data Availability

• All data reported in this paper will be shared by the lead contact upon request. • This paper does not report original code. • Any additional information required to reanalyze the data reported in this paper is available from the lead contact upon request.

## References

[bib1] Amores D.R., Warren L.A. (2007). Identifying when microbes biosilicify: the interconnected requirements of acidic pH, colloidal SiO_2_ and exposed microbial surface. Chem. Geol..

[bib2] Aubrecht R., Brewer-Carías C., Šmída B., Audy M., Kováčik Ľ. (2008). Anatomy of biologically mediated opal speleothems in the World's largest sandstone cave: cueva Charles Brewer, Chimantá Plateau, Venezuela. Venezuela. Sediment. Geol..

[bib3] Baker A., Genty D., BAker A. (1998). Environmental pressures on conserving cave speleothems: effects of changing surface land use and increased cave tourism. J. Environ. Manag..

[bib4] Baldock J.A., Oades J.M., Vassallo A.M., Wilson M.A. (1990). Solid-state CP/MAS ^13^C NMR analysis of bacterial and fungal cultures isolated from a soil incubated with glucose. Soil Res..

[bib5] Barrera V., Monteros-Altamirano Á., Valverde M., Escudero L., Allauca J., Zapata A. (2021). Characterization and classification of agricultural production systems in the Galapagos islands (Ecuador). Agric. Sci..

[bib6] Borgman P., Lopez R.D., Lane A.L. (2019). The expanding spectrum of diketopiperazine natural product biosynthetic pathways containing cyclodipeptide synthases. Org. Biomol. Chem..

[bib7] Borsato A., Frisia S., Jones B., Van der Borg K. (2000). Calcite moonmilk: crystal morphology and environment of formation in caves in the Italian Alps. J. Sediment. Res..

[bib8] Burgoyne J., Crepeau R., Jensen J., Smith H., Baker G., Leavitt S.D. (2021). Lampenflora in a show cave in the Great Basin is distinct from communities on naturally lit rock surfaces in nearby wild caves. Microorganisms.

[bib9] Cacchio P., Ferrini G., Ercole C., Del Gallo M., Lepidi A. (2014). Biogenicity and characterization of moonmilk in the grotta Nera (majella National Park, abruzzi, central Italy). J. Cave Karst Stud..

[bib10] Cailleau G., Verrecchia E.P., Braissant O., Emmanuel L. (2009). The biogenic origin of needle fibre calcite. Sedimentology.

[bib11] Cañaveras J.C., Cuezva S., Sanchez-Moral S., Lario J., Laiz L., Gonzalez J.M., Saiz-Jimenez C. (2006). On the origin of fiber calcite crystals in moonmilk deposits. Naturwissenschaften.

[bib12] Climate data of Galápagos (2021). https://pt.climate-data.org/america-do-sul/equador/provincia-de-imbabura/galapagos-178817/.

[bib13] Constantin S., Toulkeridis T., Moldovan O.T., Villacís M., Addison A. (2019). Caves and karst of Ecuador–state-of-the-art and research perspectives. Phys. Geogr..

[bib14] Cuezva S., Cañaveras C., González R., Lario J., Luque L., Sáiz-Jiménez C., Sánchez-Moral S., Soler V. (2003). Origen bacteriano de espelotemas tipo moonmilk en ambiente kárstico (Cueva de Altamira, Cantabria, España). Estud. Geol..

[bib15] Cuezva S., Fernandez-Cortes A., Porca E., Pašić L., Jurado V., Hernandez-Marine M., Serrano-Ortiz P., Hermosin B., Cañaveras J.C., Sanchez-Moral S., Saiz-Jimenez C. (2012). The biogeochemical role of Actinobacteria in altamira cave, Spain. FEMS Microbiol. Ecol..

[bib16] Davis K.J., Dove P.M., Wasylenki L.E., De Yoreo J.J. (2004). Morphological consequences of differential Mg^2+^ incorporation at structurally distinct steps on calcite. Am. Mineral..

[bib17] Daza R., Gázquez F., Miller A.Z., Sáiz-Jimenez C., Calaforra J.M., Forti P., Rull F., Medina J., Aurelio S.A., Jesus M.F., Theofilos T. (2016). Proceedings of the 17th International Symposium of Vulcan speleology. Big Island, Hawaii (USA).

[bib18] Debut A., Toulkeridis T., Vaca A.V., Arroyo C.R. (2021). Origin of color variations of thin, nano-sized layers of volcanic cinder from the Sierra Negra Volcano of the Galapagos Islands. Uniciencia.

[bib19] Dovbeshko G., Fesenko O.M., Boyko V., Boiko V.V., Romanyuk V., Moiseienko V., Moiseyenko V., Gorelik V., DoLgov L., Kiisk V., SIldos I. (2012). Vibrational spectra of opal-based photonic crystals. IOP Conf. Ser. Mater. Sci. Eng..

[bib20] Fry B. (2006). Stable isotope ecology.

[bib21] Gallardo G., Toulkeridis T. (2008).

[bib22] Gerschlauer F., Saiz G., Schellenberger Costa D., Kleyer M., Dannenmann M., Kiese R. (2019). Stable carbon and nitrogen isotopic composition of leaves, litter, and soils of various ecosystems along an elevational and land-use gradient at Mount Kilimanjaro, Tanzania. Biogeosciences.

[bib23] Gonzalez-Pimentel J.L., Martin-Pozas T., Jurado V., Miller A.Z., Caldeira A.T., Fernandez-Lorenzo O., Sanchez-Moral S., Saiz-Jimenez C. (2021). Prokaryotic communities from a lava tube cave in La Palma Island (Spain) are involved in the biogeochemical cycle of major elements. PeerJ..

[bib24] Gonzalez-Pimentel J.L., Miller A.Z., Jurado V., Laiz L., Pereira M.F.C., Saiz-Jimenez C. (2018). Yellow coloured mats from lava tubes of La Palma (Canary Islands, Spain) are dominated by metabolically active Actinobacteria. Sci. Rep..

[bib25] Granger J., Wankel S.D. (2016). Isotopic overprinting of nitrification on denitrification as a ubiquitous and unifying feature of environmental nitrogen cycling. Proc. Natl. Acad. Sci. U S A..

[bib26] Handley K.M., Turner S.J., Campbell K.A., Mountain B.W. (2008). Silicifying biofilm exopolymers on a hot-spring microstromatolite: templating nanometer-thick laminae. Astrobiology.

[bib27] Harpp K.S., Hall P.S., Jackson G., Harpp K.S., Mittelstaedt E., D'Ozouville N., Graham D.W. (2014). The Galapagos: a natural laboratory for the earth sciences.

[bib28] Hatcher P.G., Spiker E.C., Szeverenyi N.M., Maciel G.E. (1983). Selective preservation and origin of petroleum-forming aquatic kerogen. Nature.

[bib29] Hathaway J.J., Sinsabaugh R.L., Dapkevicius M.D.L.N.E., Northup D.E. (2014). Diversity of ammonia oxidation (amoA) and nitrogen fixation (nifH) genes in lava caves of Terceira, Azores, Portugal. Geomicrobiol. J..

[bib30] Hernández J., Izquierdo I., Oromí P. (1992). Catálogo espeleológico de las Islas Galápagos.

[bib31] Hill C., Forti P. (1997).

[bib32] Howell N.K., Arteaga G., Nakai S., Li-chan E.C.Y. (1999). Raman spectral analysis in the C - H stretching region of proteins and amino acids for investigation of hydrophobic interactions. J. Agric. Food Chem..

[bib33] Huber K.J., Geppert A.M., Wanner G., Fösel B.U., Wüst P.K., Overmann J. (2016). The first representative of the globally widespread subdivision 6 Acidobacteria, *Vicinamibacter silvestris* gen. nov., sp. nov., isolated from subtropical savannah soil. Int. J. Syst. Evol. Microbiol..

[bib34] Jiménez-Morillo N.T., Almendros G., De la Rosa J.M., Jordán A., Zavala L.M., Granged A.J.P., González-Pérez J.A., González-Pérez J.A. (2020). Effect of a wildfire and of post-fire restoration actions in the organic matter structure in soil fractions. Sci. Total Environ..

[bib35] Jiménez-Morillo N.T., Almendros G., González-Vila F.J., Jordán A., Zavala L.M., de la Rosa J.M., González-Pérez J.A. (2020). Fire effects on C and H isotopic composition in plant biomass and soil: bulk and particle size fractions. Sci. Total Environ..

[bib36] Jiménez-Morillo N.T., De la Rosa J.M., Waggoner D., Almendros G., González-Vila F.J., González-Pérez J.A. (2016). Fire effects in the molecular structure of soil organic matter fractions under *Quercus suber* cover. Catena.

[bib37] Jiménez-Morillo N.T., González-Pérez J.A., Almendros G., De la Rosa J.M., Waggoner D.C., Jordán A., Zavala L.M., González-Vila F.J., Hatcher P.G. (2018). Ultra-high resolution mass spectrometry of physical speciation patterns of organic matter in fire-affected soils. J. Environ. Manage..

[bib38] Jones B. (2009). Cave Pearls—the integrated product of abiogenic and biogenic processes. J. Sediment. Res..

[bib39] Jones B., Renaut R.W. (1996). Influence of thermophilic bacteria on calcite and silica precipitation in hot springs with water temperatures above 90 °C: evidence from Kenya and New Zealand. Can. J. Earth Sci..

[bib40] Kempe S.F., Middleton G., Addison A., Toulkeridis T., Hoese G. (2021). New insights into the genesis of pyroducts of the Galápagos islands, Ecuador. Acta. Carsol..

[bib41] Kramer R.W., Kujawinski E.B., Hatcher P.G. (2004). Identification of black carbon derived structures in a volcanic ash soil humic acid by fourier transform ion cyclotron resonance mass spectrometry. Environ. Sci. Technol..

[bib42] Kuhnle G.G., Joosen A.M.C.P., Kneale C.J., O'Connell T.C. (2013). Carbon and nitrogen isotopic ratios of urine and faeces as novel nutritional biomarkers of meat and fish intake. Eur. J. Nutr..

[bib43] Lacelle D., Lauriol B., Clark I.D. (2004). Seasonal isotopic imprint in moonmilk from Caverne de l’Ours (Quebec, Canada): implications for climatic reconstruction. Can. J. Earth Sci..

[bib44] Li X., Chevez T., De Silva A.O., Muir D.C.G., Kleywegt S., Simpson A., Simpson M.J., Jobst K.J. (2021). Which of the (mixed) halogenated n-alkanes are likely to be persistent organic pollutants?. Environ. Sci. Technol..

[bib45] Liu Z., Sleighter R.L., Zhong J., Hatcher P.G. (2011). The chemical changes of DOM from black waters to coastal marine waters by HPLC combined with ultrahigh resolution mass spectrometry. Estuar. Coast Shelf Sci..

[bib46] López-Martínez R., Barragán R., Beraldi-Campesi H., Lánczos T., Vidal-Romaní J., Aubrecht R., Bernal Uruchurtu J.P., Pi Puig T., Espinasa-Pereña R., Espinasa-Pereña R. (2016). Morphological and mineralogical characterization of speleothems from the Chimalacatepec lava tube system, Central Mexico. Int. J. Speleol..

[bib47] Maciejewska M., Całusińska M., Cornet L., Adam D., Pessi I.S., Malchair S., Delfosse P., Baurain D., Barton H., Carnol M., Rigali S. (2018). High-throughput sequencing analysis of the actinobacterial spatial diversity in moonmilk deposits. Antibiotics.

[bib48] Maciejewska M., Pessi I.S., Arguelles-Arias A., Noirfalise P., Luis G., Ongena M., Barton H., Carnol M., Rigali S. (2015). Streptomyces lunaelactis sp. nov., a novel ferroverdin A-producing Streptomyces species isolated from a moonmilk speleothem. Antonie Leeuwenhoek.

[bib49] Martínez-Arkarazo I., Angulo M., Zuloaga O., Usobiaga A., Madariaga J.M. (2007). Spectroscopic characterisation of moonmilk deposits in pozalagua tourist cave (karrantza, Basque country, North of Spain). Spectrochim. Acta Part A Mol. Biomol. Spectrosc..

[bib50] Melim L.A., Northup D.E., Spilde M.N., Boston P.J. (2015). Update: living reticulated filaments from herbstlabyrinth-adventhöhle cave system, Germany. J. Cave Karst Stud..

[bib51] Melim L.A., Northup D.E., Spilde M.N., Jones B., Boston P.J., Bixby R.J. (2008). Reticulated filaments in cave pool speleothems: microbe or mineral?. J. Cave. Karst. Stud..

[bib52] Michel A.J., Ward L.M., Goffredi S.K., Dawson K.S., Baldassarre D.T., Brenner A., Gotanda K.M., McCormack J.E., Mullin S.W., O’Neill A. (2018). The gut of the finch: uniqueness of the gut microbiome of the Galápagos vampire finch. Microbiome.

[bib53] Miller A.Z., De la Rosa J.M., Jiménez-Morillo N.T., Pereira M.F.C., González-Pérez J.A., González-Pérez J.A., Calaforra J.M., Saiz-Jimenez C. (2016). Analytical pyrolysis and stable isotope analyses reveal past environmental changes in coralloid speleothems from Easter Island (Chile). J. Chromatogr. A..

[bib54] Miller A.Z., De la Rosa J.M., Jiménez-Morillo N.T., Pereira M.F.C., Gonzalez-Perez J.A., Knicker H., Saiz-Jimenez C. (2020). Impact of wildfires on subsurface volcanic environments: new insights into speleothem chemistry. Sci. Total Environ..

[bib55] Miller A.Z., García-Sánchez A.M., L Coutinho M., Costa Pereira M.F., Gázquez F., Calaforra J.M., Forti P., Martínez-Frías J., Toulkeridis T., Caldeira A.T., Saiz-Jimenez C. (2020). Colored microbial coatings in show caves from the Galapagos islands (Ecuador): first microbiological approach. Coatings.

[bib56] Miller A.Z., Garcia-Sanchez A.M., Martin-Sanchez P.M., Costa Pereira M.F., Spangenberg J.E., Jurado V., Dionísio A., Afonso M.J., Iglé sias Chaminé H.I., Hermosin B., Saiz-Jimenez C. (2018). Origin of abundant moonmilk deposits in a subsurface granitic environment. Sedimentology.

[bib57] Miller A.Z., Hernández-Mariné M., Jurado V., Dionísio A., Barquinha P., Fortunato E., Afonso M.J., Afonso M.J., Chaminé H.I., Saiz-Jimenez C., Dionisio A. (2012). Enigmatic reticulated filaments in subsurface granite. Environ. Microbiol. Rep..

[bib58] Miller A.Z., Pereira M.F., Calaforra J.M., Forti P., Dionísio A., Dionísio A., Saiz-Jimenez C. (2015). Ana Heva lava tube (Easter Island, Chile): preliminary characterization of the internal layers of coralloid-type speleothems. Microsc. Microanal..

[bib59] Miller A.Z., Pereira M.F.C., Calaforra J.M., Forti P., Dionísio A., Saiz-Jimenez C. (2014). Siliceous speleothems and associated microbe-mineral interactions from Ana Heva lava tube in Easter Island (Chile). Geomicrobiol. J..

[bib60] Mohapatra B., Phale P.S. (2021). Microbial degradation of naphthalene and substituted naphthalenes: metabolic diversity and genomic insight for bioremediation. Front. Bioeng. Biotechnol..

[bib61] Napieralski S.A., Roden E.E. (2020). The weathering microbiome of an outcropping granodiorite. Front. Microbiol..

[bib62] Northup D.E., Melim L.A., Spilde M.N., Hathaway J.J.M., Garcia M.G., Moya M., Stone F.D., Boston P.J., Dapkevicius M.L.N.E., Riquelme C. (2011). Lava cave microbial communities within mats and secondary mineral deposits: implications for life detection on other planets. Astrobiology.

[bib63] Park S., Cho Y.-J., Jo K., Lee E.-J., Lee J.-S. (2020). Microbial diversity in moonmilk of baeg-nyong cave, Korean CZO. Front. Microbiol..

[bib64] Parker J.E., Thompson S.P., Lennie A.R., Potter J., Tang C.C. (2010). A study of the aragonite-calcite transformation using Raman spectroscopy, synchrotron powder diffraction and scanning electron microscopy. Cryst. Eng. Comm..

[bib65] Perzborn M., Syldatk C., Rudat J. (2013). Enzymatical and microbial degradation of cyclic dipeptides (diketopiperazines). Amb. Express.

[bib66] Polyak V.J., Provencio P.P., Hildreth-Werker A.L., Werker J.C. (2006). Cave Conservation and Restoration.

[bib67] Rabbi S.M.F., Wilson B.R., Lockwood P.V., Daniel H., Young I.M. (2014). Soil organic carbon mineralization rates in aggregates under contrasting land uses. Geoderma.

[bib68] Richter D.K., Neuser R.D., Schreuer J., Gies H., Immenhauser A. (2011). Radiaxial-fibrous calcites: a new look at an old problem. Sediment. Geol..

[bib69] Riquelme C., Marshall Hathaway J.J., Enes Dapkevicius M.d.L.N., Miller A.Z., Kooser A., Northup D.E., Jurado V., Fernandez O., Saiz-Jimenez C., Cheeptham N. (2015). Actinobacterial diversity in volcanic caves and associated geomicrobiological interactions. Front. Microbiol..

[bib70] Sanchez-Moral S., Portillo M.C., Janices I., Cuezva S., Fernández-Cortés A., Cañaveras J., Gonzalez J.M. (2012). The role of microorganisms in the formation of calcitic moonmilk deposits and speleothems in Altamira Cave. Geomorphology.

[bib71] Sauro F., Cappelletti M., Ghezzi D., Columbu A., Hong P.Y., Zowawi H.M., Carbone C., Piccini L., Vergara F., Zannoni D., De Waele J. (2018). Microbial diversity and biosignatures of amorphous silica deposits in orthoquartzite caves. Sci. Rep..

[bib72] Schwartz D. (2014).

[bib73] Simkin T. (1984). Geology of Galapagos. Biol. J. Linn. Soc..

[bib74] Sodo A., Casanova Municchia A., Barucca S., Bellatreccia F., Della Ventura G., Butini F., Ricci M.A. (2016). Raman, FT-IR and XRD investigation of natural opals. J. Raman Spectrosc..

[bib75] Stoops G.J. (1976). On the nature of "lublinite" from Hollanta (Turkey). Am. Mineral..

[bib76] Suchý V., Borecká L., Pachnerová Brabcová K., Havelcová M., Svetlik I., Machovič V., Lapčák L., Ovšonková Z.A. (2021). Microbial signatures from speleothems: a petrographic and scanning electron microscopy study of coralloids from the Koněprusy Caves (the Bohemian Karst, Czech Republic). Sedimentology.

[bib77] Sutton R., Sposito G. (2005). Molecular structure in soil humic substances: the new view. Environ. Sci. Technol..

[bib78] Szpak P. (2014). Complexities of nitrogen isotope biogeochemistry in plant–soil systems: implications for the study of ancient agricultural and animal management practices. Front. Plant. Sci..

[bib79] Taylor S.J., Addison A., Toulkeridis T. (2011). Biological potential of under-studied cave fauna of the Galapagos Islands. Rev. Geoespacial..

[bib80] Urbani F., Compère P., Willems L. (2005). Opal-A speleothems of wei-assipu-tepui, roraima province, Brazil. Bol. la Soc. Venez. Espeleol..

[bib81] Vanghi V., Frisia S., Borsato A. (2017). Genesis and microstratigraphy of calcite coralloids analysed by high resolution imaging and petrography. Sediment. Geol..

[bib82] Vidal-Romaní J.R., Sanjurjo Sánchez J., Rodríguez M.V., Fernández Mosquera D. (2010). Speleothem development and biological activity in granite cavities. Geomorphol. Reli. Process. Environ..

[bib83] Vieira S., Luckner M., Wanner G., Overmann J. (2017). Luteitalea pratensis gen. nov., sp. nov. a new member of subdivision 6 Acidobacteria isolated from temperate grassland soil. Int. J. Syst. Evol. Microbiol..

[bib84] Violette S., d’Ozouville N., Pryet A., Deffontaines B., Fortin J., Adelinet M., Karen S.H., Mittelstaedt E., D'Ozouville N., Graham D.W. (2014). The Galápagos: A Natural laboratory for the Earth sciences, Geophysical.

[bib85] Wang A., Freeman J.J., Jolliff B.L. (2015). Understanding the Raman spectral features of phyllosilicates. J. Raman Spectrosc..

[bib86] Webb J.A., Finlayson B.L. (1987). Incorporation of Al, Mg, and water in opal-A: evidence from speleothems. Am. Mineral..

[bib87] Westall F., de Ronde C.E.J., Southam G., Grassineau N., Colas M., Cockell C., Lammer H. (2006). Implications of a 3.472–3.333 Gyr-old subaerial microbial mat from the Barberton greenstone belt, South Africa for the UV environmental conditions on the early Earth. Phil. Trans. R. Soc. B..

[bib88] Westall F., Foucher F., Bost N., Bertrand M., Loizeau D., Vago J.L., Kminek G., Gaboyer F., Campbell K.A., Bréhéret J.G. (2015). Biosignatures on Mars: what, where, and how? Implications for the search for martian life. Astrobiology.

[bib89] White W.B. (2010). Secondary minerals in volcanic caves: data from Hawai'i. J. Cave Karst. Stud..

[bib90] White W.M., McBirney A.R., Duncan R.A. (1993). Petrology and geochemistry of the Galápagos Islands: portrait of a pathological mantle plume. J. Geophys. Res. Solid Earth.

[bib91] Wray R.A.L. (1999). Opal and chalcedony speleothems on quartz sandstones in the Sydney region, southeastern Australia. Aust. J. Earth Sci..

[bib92] Wray R.A.L., Sauro F. (2017). An updated global review of solutional weathering processes and forms in quartz sandstones and quartzites. Earth Sci. Rev..

[bib93] Wynne J.J., Howarth F.G., Mammola S., Ferreira R.L., Cardoso P., Lorenzo T.D., Galassi D.M.P., Medellin R.A., Miller B.W., Sánchez-Fernández D. (2021). A conservation roadmap for the subterranean biome. Conserv. Lett..

[bib94] Zhang C., Tayyab M., Abubakar A.Y., Yang Z., Pang Z., Islam W., Lin Z., Li S., Luo J., Fan X. (2019). Bacteria with different assemblages in the soil profile drive the diverse nutrient cycles in the sugarcane straw retention ecosystem. Diversity.

[bib95] Zulkarami B., Ashrafuzzaman M., Husni M.O., Ismail M.R. (2011). Effect of pyroligneous acid on growth, yield and quality improvement of rockmelon in soilless culture. Aust. J. Crop. Sci..

