# Supplementary material for: Organic geochemistry and mineralogy suggest anthropogenic impact in speleothem chemistry from volcanic show caves of the Galapagos
Source: iScience. 2022 Jun 9;25(7):104556. doi: 10.1016/j.isci.2022.104556 (PMC9250005; doi:10.1016/j.isci.2022.104556)
Supplement: Document S1. Figures S1–S5 and Tables S1 and S2 [file mmc1.pdf]

iScience, Volume *25*

Supplemental information

Organic geochemistry and mineralogy suggest anthropogenic impact in speleothem chemistry from volcanic show caves of the Galapagos

Ana Z. Miller, Nicasio T. Jiménez-Morillo, Mathilda L. Coutinho, Fernando Gazquez, Vera Palma, Francesco Sauro, Manuel F.C. Pereira, Fernando Rull, Theofilos Toulkeridis, Ana T. Caldeira, Paolo Forti, and José M. Calaforra

Organic geochemistry and mineralogy suggest anthropogenic impact in speleothem chemistry from volcanic show caves of the Galapagos

Ana Z. Miller,[1,2,10*] Nicasio T. Jiménez-Morillo,[3] Mathilda L. Coutinho,[2] Fernando Gazquez,[4,5] Vera Palma,[2] Francesco Sauro,[6] Manuel F.C. Pereira,[7] Fernando Rull,[8] Theofilos Toulkeridis,[9] Ana T. Caldeira,[2] Paolo Forti,[6] José M. Calaforra[4,5]

[1]Instituto de Recursos Naturales y Agrobiologia de Sevilla (IRNAS-CSIC), Sevilla, Spain

[2]HERCULES Laboratory, University of Évora, Évora, Portugal

[3]MED—Mediterranean Institute for Agriculture, Environment and Development, Universidade de Évora, Portugal

[4]Department of Biology and Geology, Universidad de Almeria, Spain

[5]Andalusian Centre for the Monitoring and Assessment of Global Change (CAESCG), University of Almería, Spain

[6]Department of Earth Sciences and Environmental Geology, University of Bologna, Bologna, Italy

[7]CERENA, Instituto Superior Técnico, Universidade de Lisboa, Portugal

[8]CSIC-CAB Associated Unit ERICA, Department of Condensed Matter Physics, Mineralogy and Crystallography, University of Valladolid, Boecillo, Spain

[9]Universidad de las Fuerzas Armadas (ESPE), Campus Sangolquí, Ecuador

[10]Lead contact

*Correspondence: anamiller@irnas.csic.es / anamiller@uevora.pt

Supplemental information

Supplemental Figures

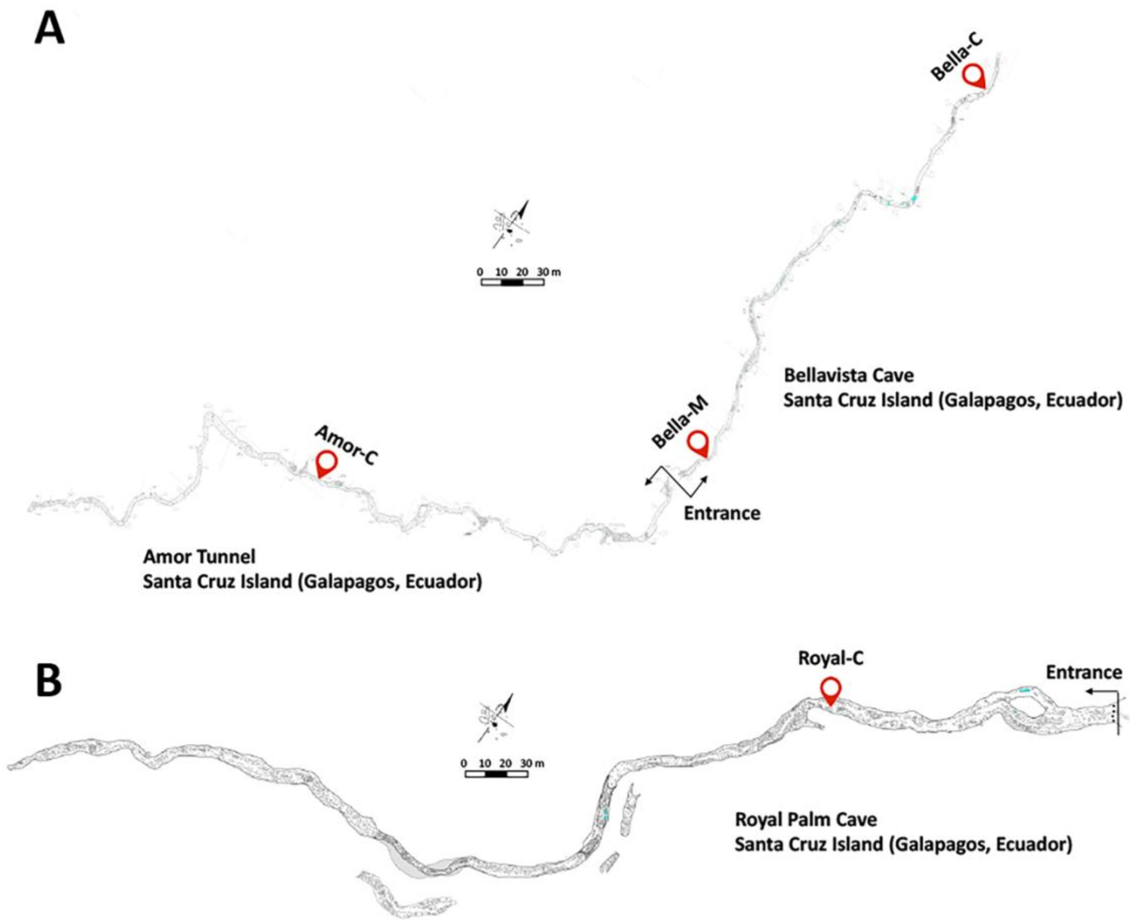

Figure S1. Topographical maps of the two lava tubes studied from Santa Cruz Island (Galapagos, Ecuador) with indication of the sampling points: (A) Bellavista Cave and Amor Tunnel section; (B) Royal Palm Cave. Related to STAR Methods.

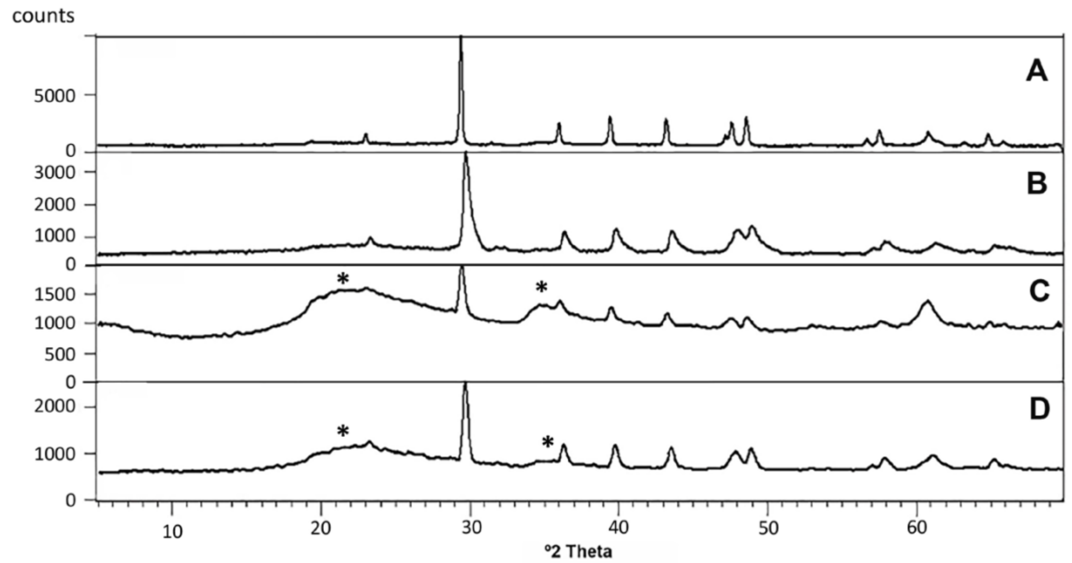

Figure S2. X-Ray diffraction patterns of the studied speleothems from lava tubes of Santa Cruz Island (Galapagos, Ecuador): (A) Bella-M (moonmilk); (B) Bella-C (coralloids); (C) Amor-C (coralloids); (D) Royal-C (coralloids); * – amorphous compound. Related to Figures 1 – 4.

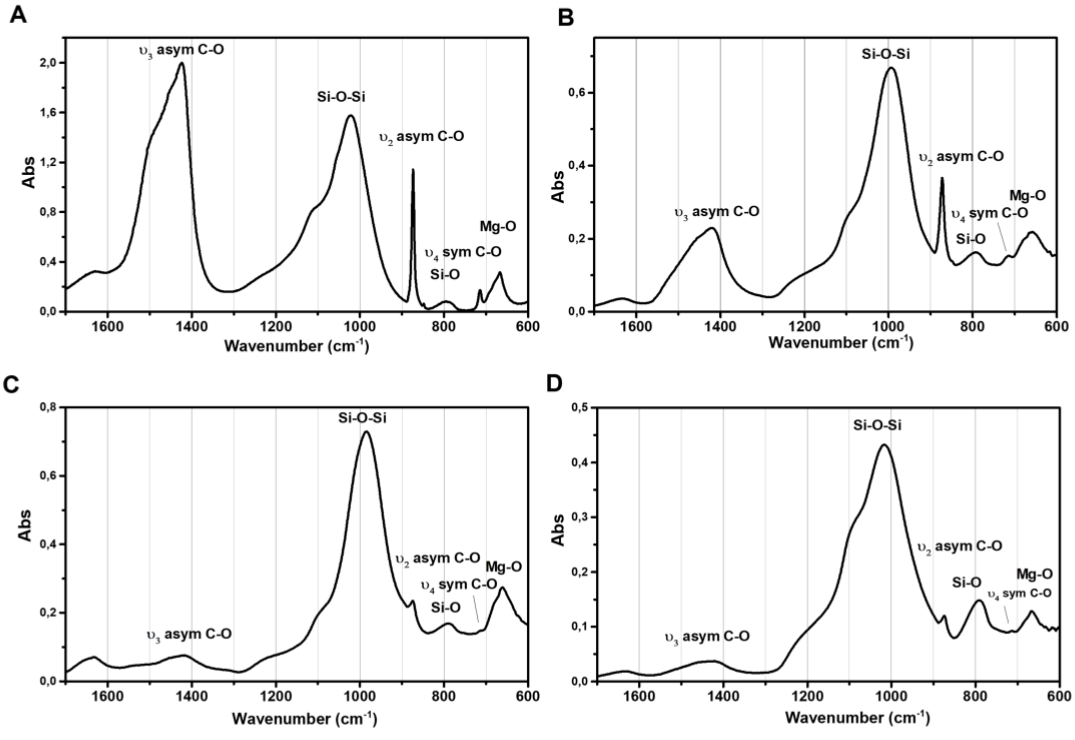

Figure S3. Infrared spectra of the studied speleothems from lava tubes of Santa Cruz Island (Galapagos, Ecuador): (A) Bella-M (moonmilk); (B) Bella-C (coralloids); (C) Amor-C (coralloids); (D) Royal-C (coralloids), showing the characteristic IR spectra of calcite, opal-A and magnesium oxide. Related to Figures 1 – 4.

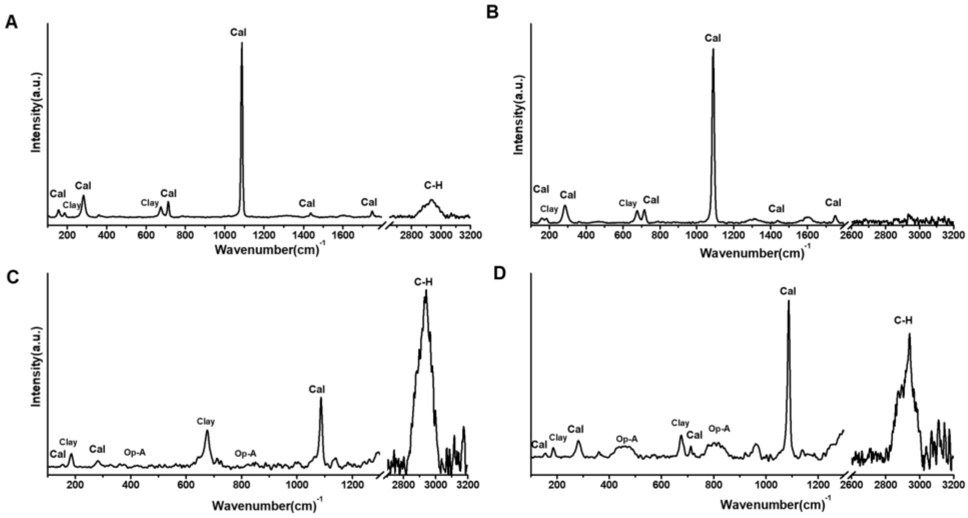

Figure S4. Raman spectra of the studied speleothems from lava tubes of Santa Cruz Island (Galapagos, Ecuador): (A) Bella-M (moonmilk); (B) Bella-C (coralloids); (C) Amor-C (coralloids); (D) Royal-C (coralloids). Cal = calcite; Clay = montmorillonite, Op-A = opal-A. Related to Figures 1 – 4.

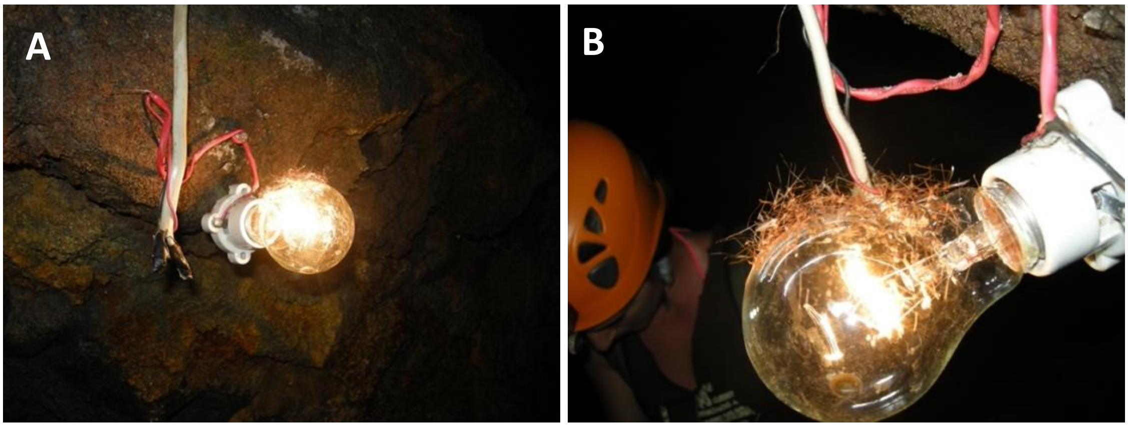

Figure S5. Royal Palm Cave lighting system. A) Electrical system installation; and B) Dead insects on the lamp. Related to Figure 6.

Supplementary Tables

Table S1. Interpretation of band positions in FTIR and Raman spectrum of the speleothems. Related to Figures 1 – 4.

IR (cm^{-1})	Assignment	Possible mineral phase	Speleothem	Ref.	Raman (cm^{-1})	Assignment	Possible mineral phase	Speleothem	Ref.
660-667	Mg-O	Sepiolite	Bella-M, Bella- C, Amor-C, Royal-C	Miller et al. 2014	154-157	Lattice mode calcite	Calcite	Bella-M, Bella- C, Amor-C, Royal-C	Parker et al. 2010
711-716	O-C-O bending (in-plane deformation)	Calcite	Bella-M, Bella- C, Amor-C, Royal-C	Miller et al. 2018	186-188	Lattice mode clay	Clay mineral (montmorillonite)	Bella-M, Bella- C, Amor-C, Royal-C	Wang et al. 2015
791-797	Si-O s-stretch	Opal A	Bella-M, Bella- C, Amor-C, Royal-C	Miller et al. 2014; Sodo et al. 2016	282-286	Lattice mode calcite	Calcite	Bella-M, Bella- C, Amor-C, Royal-C	Parker et al. 2010
870-875	O-C-O bending (out-plane deformation)	Calcite	Bella-M, Bella- C, Amor-C, Royal-C	Miller et al. 2018	674-678	Si-O-Si vibrations	Clay mineral (trioctahedral mineral)	Bella-M, Bella- C, Amor-C, Royal C	Wang et al. 2015
983-1100	Si-O a-stretch (SiO_4)	Opal A	Bella-M, Bella- C, Amor-C, Royal-C	Miller et al. 2014; Sodo et al. 2016	713-715	Sym. in-plane bending C-O	Calcite	Bella-M, Bella- C,	Parker et al. 2010
1420-1430	C-O a-stretch	Calcite	Bella-M, Bella- C, Amor-C, Royal-C	Miller et al. 2018	800	Si-O-Si vibrations	Opal-A	Amor-C, Royal-C	Dovbeshko et al. 2004, Sodo et al. 2016
					960	Silanol group (Si-OH)	Opal-A	Amor-C, Royal-C	Sodo et al. 2016
					1086-1089	C-O s-stretch	Calcite	Bella-M, Bella- C, Amor-C, Royal-C	Parker et al. 2010
					1318-1435	C-O a-stretch	Calcite	Bella-M, Bella- C,	Parker et al. 2010
					1602-1748	C-O a-stretch	Calcite	Bella-M, Bella- C,	Parker et al. 2010
					2934-2940	C-H vibrations	Organic compounds	Bella-M, Amor6, Royal-C	Howell et al. 1999

Table S2. List of compounds identified by Py-GC/MS in speleothem samples. Related to Figure 6.

Family	RT (min)	Name of compound	Bella M %	Bella C %	Amor C %	Royal C %
**Alkyl hallogen**	8.6684	Alkyl hallogen	-	-	-	+
	9.2939	Alkyl hallogen	-	-	-	+
	10.0207	Alkyl hallogen	-	-	-	+
	10.5678	Alkyl hallogen	-	-	-	+
	10.9051	Alkyl hallogen	-	-	-	+
**Aromatic**	3.0540	Toluene	++	++	-	-
	3.6307	*o*-Xylene	-	++	-	-
	3.6935	1,2-Dihydrocatechol	-	-	+	-
	3.7782	Styrene	++	++	+	-
	4.2587	Benzene, 1-ethyl-2methyl	-	-	-	++
	4.4923	Benzene, 1,2,3-trimethyl	+	++	-	+
	5.2083	Benzenamine, 2-methoxy-	-	-	++	-
	5.4155	*o*-Cymene	+	+	-	-
	5.5271	Benzene, 1,2,4,5-tetramethyl-	++	++	-	++
	5.7315	Benzene, 1-methyl-2-(2-propenyl)	-	+	-	-
	5.8371	*p*-cymene	++	++	-	++
	6.1950	3,4-Dimethylcumene	++	-	-	-
	7.0036	Benzene, pentamethyl	++	+	-	+
**Condensed compound**	6.1994	Naphthalene	-	++	-	-
	6.8773	Naphthalene, 1,2-dihydro-6-methyl-	-	+	-	-
	7.2069	Naphthalene, 1-methyl-	-	+	+	+
	7.3765	Naphthalene, 2-methyl-	+	+	-	+
	8.3494	Naphthalene, 1,3-dimethyl-	-	++	-	+
	9.2886	Naphthalene, 2,3,6-trimethyl	-	++	+	-
	10.6747	Chamazulene	-	+	-	-
**Lipid**	2.0115	Propanal, 2-methyl	+	+++	++	-
	2.0189	2-Pentene, 4-methyl	-	-	-	+
	2.3276	Acetic acid	-	-	++	-
	2.4434	Butanal, 3-methyl	++	+++	+++	-
	2.4457	Cyclopentene, 4-methyl-	-	-	-	++
	2.5845	2-Propanone, 1-hydroxyl	-	-	+	-
	2.6075	Cyclopentane, 1,2-dimethyl	+	-	-	-
	2.6855	3-Hexyne, 2-methyl	-	+++	-	+++
	2.9467	Cyclopropene, 3,3-diethyl	++	+++	-	+++
	3.1727	4-Octene	+	++	-	+++
	3.2849	Heptane, 2,4-dimethyl-	-	++	-	-
	3.4630	Cyclopentane, butyl	-	-	-	+++
	3.7341	Cyclohexane, 1,2,3-trimethyl	-	-	-	+++
	3.8599	Cyclopentene, 1-(1-methylethyl)	+	-	-	-
	3.9994	5-Decene-	-	-	-	+
	4.1617	Nonane, 2-methyl	-	-	-	+
	4.1619	Hexane, 3-ethyl-3-methyl	-	+	-	-
	4.2572	*trans*-3-Decene	-	-	-	++
	4.2638	Heptane, 2,6-dimethyl-	+	-	-	-
	4.3737	1-Decene	+	-	+	-
	4.4049	Decane	-	+	-	+
	4.5806	Decane, 4-methyl	+	+	-	-
	4.5845	Octane, 3,3-dimethyl	-	-	-	+
	4.8709	Decane, 3,8-dimethyl-	+++	-	-	+++
	4.8533	*L*-Linalool	-	-	+	-
	4.8711	Heptane, 2,5,5-trimethyl	-	+++	-	-
	5.0333	Borneol	-	-	++	-
	5.1369	2-Dodecene	+	++	-	+++
	5.2221	Decane, 3,7-dimethyl-	+	-	-	-
	5.9848	4-Dodecene	+	-	-	-
	6.0467	Dodecane	-	-	+	+++
	6.6338	Dodecane, 4,6-dimethyl-	+++	++	-	++
	6.7975	Dodecane, 2,6,11-trimethyl-	+++	+	-	-
	6.8808	1-Tridecene	-	-	+	-
	6.9460	Tridecane	-	-	+	-
	7.2927	Nonane, 3-methyl-5-propyl	+	-	-	-
	7.4663	3-Methyl-1-(tetraohydropyrrolo(1,2-c)oxazol-3-ylidene)-butan-2-one	-	-	+	-
	7.5285	Eicosene, 10-methyl	-	-	-	+
	7.7856	1-Tetradecene	-	-	+	-
	7.8576	Tetradecane	++	+	+	+
	8.4295	Undecane, 4,7-dimethyl	+++	-	-	-
	8.5448	Pentadecane, 2,6,10-trimethyl	+	-	-	-
	8.6720	Heptadecane, 2,6,10,15-tetramethyl	++	-	-	-

Continued

Family	*RT (min)*	*Name of compound*	*%*	*%*	*%*	*%*
**Lipid**	8.7531	Pentadecane	+++	+	-	+++
	9.1373	Branched Alkane	++	+	-	-
	9.1349	Branched Alkane	-	-	-	+
	9.2991	Branched Alkane	++	-	-	-
	9.6067	Hexadecane	++	-	++	+
	9.6892	Branched Alkane	++	-	-	-
	9.9453	Branched Alkane	++	-	-	-
	10.0253	Branched Alkane	++	-	+	-
	10.1251	Branched Alkane	+	-	-	-
	10.2815	Branched Alkane	++	-	-	-
	10.3596	Branched Alkane	+	-	-	-
	10.4322	Heptadecane	+++	-	++	+
	10.5715	Branched Alkane	+++	-	-	-
	10.7400	Branched Alkane	+	-	-	-
	10.9084	Branched Alkane	+++	-	-	-
	11.0872	Branched Alkane	+	-	-	-
	11.2243	Octadecane	++	-	-	-
	11.6447	Branched Alkane	++	-	++	+
	11.9512	Branched Alkane	++	-	-	-
	12.1569	Branched Alkane	+	-	-	-
	12.2490	Branched Alkane	++	-	-	-
	12.5068	Branched Alkane	+	-	-	-
	12.5868	Nonadecene	+	-	+	-
**Nitrogen compound**	2.1141	Pentanenitrile	-	-	+	-
	2.8169	Butanenitrile, 3-methyl	-	-	++	-
	2.8218	1-Allylazetidine	+	-	-	-
	3.1028	Acetamide	-	-	+++	-
	3.4083	2-Cyanohex-3-ene	-	-	+++	-
	4.6999	Benzamine, *N*-ethyl	-	-	+	-
	5.3533	2-Pentonenitrile, 4,4-dimethyl	-	-	+	-
	5.6637	Benzyl nitrile	+	-	+	-
	6.3511	Diisopropylaminoacetonitrile	-	-	+	-
	6.5697	Benzenepropanenitrile	-	-	+	-
	7.3669	Cyclohexanamine, *N*-cyclohexyl-*N*-methyl	-	-	+	-
	8.4045	*N*-(3-Amino-2-hydroxy-phenyl)-acetamide	-	-	++	-
	12.0544	Hexadecanenitrile	-	-	+	-
**Peptide**	2.9442	1H-Pyrrole	-	-	++	-
	3.4065	1,3-Diazine	++	-	-	-
	3.4184	1H-Pyrrole, 3-methyl	-	+++	-	-
	3.5894	Pyridine, 4-methyl-	-	-	+	-
	3.9308	1H-Pyrrole, 1-ethyl	-	++	++	-
	4.0099	Pyridine, 2,5-dimethyl	+	-	-	-
	4.0676	3-Cyclohexene-1-carboxaldehyde, 1-methyl-	+	-	-	-
	4.1248	3-Pyridinol, 2-nitro	+	-	-	-
	4.1281	2-Pyridinecarboxylic acid, 6-methyl	-	-	+	-
	4.5692	1H-Pyrrole, 2,3,5-trimethyl-	-	-	++	-
	5.4210	2,5-Pyrrolidinedione	-	-	+	-
	5.5001	1H-Pyrrole, 3-ethyl-2,4,5-trimethyl-	-	-	+	-
	5.7318	1H-Indene, 2,3-dihydro-4-methyl	-	-	-	+
	5.8382	1H-Indene, 3-methyl	-	-	+	-
	6.2127	5-*sec*-Butyluracil	-	-	+	-
	6.7823	5-Hydroxy-indan-2-one	-	-	+	-
	7.1082	Indole	-	-	+	-
	7.6272	Quinoline, 4-methyl	-	-	+	-
	7.9615	Tryptamine	-	+	-	-
	7.9726	1H-Indole, 7-methyl-	+	-	++	-
	8.0549	Diketopyperazine	-	+	-	-
	8.5019	2-(Methoxycarbonyl)-2-methyl-3-(*N*-pyrrolidino)(2H)azirine	-	-	+	-
	8.6886	Pyridine, 4-phenyl-	-	-	++	-
	8.8508	1H-Indole, 1,2-dimethyl	-	-	+	-
	9.6159	Diketopyperazine	-	+	-	-
	10.0278	Diketopyperazine	-	+	-	-
	10.8699	*N*-(Dimethylthiophosphinyl)-3-aminopyridine	-	-	+	-
**Polysaccharide**	2.2024	Furan, 2-methyl-	++	+++	+	+++
	2.6882	Furan, 2,5-dimethyl-	+	-	+	-
	3.2520	Furfural	-	-	++	-
	4.2768	2-Cyclopenten-1-one, 3-methyl	-	-	++	-

* + = Low relative abundance; ++ = medium relative abundance; +++ = high relative abundance; and - = null
